# Acetylated tau inhibits chaperone-mediated autophagy and promotes tau pathology propagation in mice

**DOI:** 10.1038/s41467-021-22501-9

**Published:** 2021-04-14

**Authors:** Benjamin Caballero, Mathieu Bourdenx, Enrique Luengo, Antonio Diaz, Peter Dongmin Sohn, Xu Chen, Chao Wang, Yves R. Juste, Susanne Wegmann, Bindi Patel, Zapporah T. Young, Szu Yu Kuo, Jose Antonio Rodriguez-Navarro, Hao Shao, Manuela G. Lopez, Celeste M. Karch, Alison M. Goate, Jason E. Gestwicki, Bradley T. Hyman, Li Gan, Ana Maria Cuervo

**Affiliations:** 1 Department of Developmental and Molecular Biology, Albert Einstein College of Medicine, Bronx, NY USA; 2 Institute for Aging Studies, Albert Einstein College of Medicine, Bronx, NY USA; 3 Institute Teofilo Hernando for Drug Discovery, Department of Pharmacology, School of Medicine, Universidad Autonoma de Madrid, Madrid, Spain; 4 Instituto de Investigación Biosanitaria Hospital de la Princesa, Madrid, Spain; 5 Helen and Robert Appel Alzheimer’s Disease Research Institute, Weill Cornell Medicine, New York, NY USA; 6 Massachusetts General Hospital, Harvard Medical School, Boston, MA USA; 7 German Center for Neurodegenerative Diseases (DZNE), Berlin, Germany; 8 Institute for Neurodegenerative Disease, University of California at San Francisco, San Francisco, CA USA; 9 Department of Psychiatry, Washington University, St. Louis, MO USA; 10 Department of Neuroscience, Icahn School of Medicine at Mount Sinai, New York, NY USA; 11 Present Address: Roche Chile Pharmaceuticals, Las Condes, Region Metropolitana Chile; 12 Present Address: Institut des Maladies Neurodégénératives, CNRS, Université de Bordeaux, Bordeaux, France; 13 Present Address: Instituto Ramón y Cajal de Investigaciones Sanitarias Hospital Ramón y Cajal, Madrid, Spain

**Keywords:** Chaperone-mediated autophagy, Cellular neuroscience, Neurodegeneration, Neural ageing

## Abstract

Disrupted homeostasis of the microtubule binding protein tau is a shared feature of a set of neurodegenerative disorders known as tauopathies. Acetylation of soluble tau is an early pathological event in neurodegeneration. In this work, we find that a large fraction of neuronal tau is degraded by chaperone-mediated autophagy (CMA) whereas, upon acetylation, tau is preferentially degraded by macroautophagy and endosomal microautophagy. Rerouting of acetylated tau to these other autophagic pathways originates, in part, from the inhibitory effect that acetylated tau exerts on CMA and results in its extracellular release. In fact, experimental blockage of CMA enhances cell-to-cell propagation of pathogenic tau in a mouse model of tauopathy. Furthermore, analysis of lysosomes isolated from brains of patients with tauopathies demonstrates similar molecular mechanisms leading to CMA dysfunction. This study reveals that CMA failure in tauopathy brains alters tau homeostasis and could contribute to aggravate disease progression.

## Abstract (web summary)

The tau protein has been implicated in neurodegenerative disorders and can propagate from cell to cell. Here, the authors show that tau acetylation reduces its degradation by chaperone-mediated autophagy, causing re-routing to other autophagic pathways and increasing extracellular tau release.

## Introduction

Accumulation of the microtubule-stabilizing protein tau in neurofibrillary tangles is a hallmark of tauopathies, including Alzheimer’s disease (AD) and frontotemporal lobar degeneration^1^. Tau function is negatively regulated by different post-translational modifications (i.e., phosphorylation^2–4^ and acetylation^5,6^). Acetylation has been described as a pathogenic post-translational modification of tau in brains from AD^7,8^ and related tauopathies patients^5,6,9,10^. Recently, acetylation sites have been precisely mapped onto the Cryo-EM structure of tau filaments from patients’ brain tissue^11^. Tau acetylation reduces its binding to microtubules, promotes tau fibrillization, reduces tau degradation^5,9^, and contributes to tau-mediated synaptic toxicity^12^. Although the emphasis on pathogenic tau clearance has mostly focused on aggregated tau, tau acetylation on lysine 174 (K^174^) was identified as an early modification in the soluble fraction of AD patients^13^. Reducing tau acetylation at K174 rescues cognitive deficits and tau-mediated neurodegeneration^13^, suggesting that reducing soluble forms of tau is efficient in improving cognitive function^14–16^.

Tau pathology spreads in the brain following a predictable pattern of progression^17,18^ attributed to cell-to-cell propagation of pathogenic tau^19,20^. However, as tau lacks conventional secretion signals^21,22^, the process of tau transmission between neighboring neurons is still poorly understood. Enhanced acetyltransferase activity in tauopathies has been shown to result in excess tau secretion and spreading^23^. However, whether this is a consequence of direct tau acetylation or due to acetylation of other cellular components remains unknown.

The ubiquitin-proteasome and the autophagic-lysosomal system have been implicated in tau clearance^24,25^ and both proteolytic systems have shown to be altered in neurodegenerative disorders, including AD^24,26,27^. Previous reports from our group and others have demonstrated tau degradation by macroautophagy, a process that requires the sequestration of cargo in double-membrane vesicles (autophagic vacuoles), which later fuse with lysosomes^28^. Chemical modulation of macroautophagy^29–32^ and genetic manipulation of essential macroautophagy genes^32,33^ change cellular tau levels. Interestingly, multiple lines of evidence link defective macroautophagy with AD^34–36^.

Our own in vitro studies support that tau can also undergo degradation via chaperone-mediated autophagy (CMA), a type of selective autophagy that encompasses direct transport of substrate proteins across the lysosomal membrane^37^. Selectivity of CMA is conferred by the ability of the heat shock cognate protein of 70 kDa (hsc70) to recognize a pentapeptide motif^38^ (biochemically related to the pentapeptide KFERQ) in CMA substrate proteins, and deliver them to the lysosomal surface for binding to the lysosome-associated membrane protein type 2A (L2A)^39,40^. Once substrates bind, L2A multimerizes into a translocation complex^41^, which allows access of substrate proteins into the lysosomal lumen one-by-one. Substrate entry requires a form of hsc70 residing inside lysosomes, that completes substrate translocation^42^. We have shown that tau contains several KFERQ-like motifs, necessary for its lysosomal translocation into lysosomes via CMA in vitro^29^.

Interestingly, the same pentapeptide recognized by hsc70 is also utilized by this cytosolic chaperone to deliver cytosolic proteins for degradation in late endosomes through endosomal microautophagy (e-MI)^43^. In e-MI, the late endosome membrane invaginates sequestering the hsc70/substrate complex into small microvesicles that are then degraded in the endosomal lumen^43^. Previous studies support that CMA and e-MI actively contribute to the clearance of soluble forms of tau in cultured cells^32^. In contrast, disease-related and phosphorylation-mimetic variants display, in many instances, low rates of degradation through these two types of selective autophagy, and in fact, they often exert an inhibitory effect on them^32^. However, the repercussion of acetylation of tau on its degradation through CMA and e-MI remains unknown.

In this study, we have analyzed the impact of acetylation on the degradation of tau by these three different autophagic pathways and found that acetylation reduces tau degradation through CMA. Although macroautophagy degrades this form of the protein with higher efficiency than the unmodified tau, the association of acetylated tau with lysosomes renders them inactive for CMA. We observed that this inhibition of CMA increases extracellular tau release and that tau spreading was enhanced in CMA-deficient mice. We demonstrate experimentally that tau release upon CMA blockage is a result of its rerouting to late endosomes via e-MI. We propose that the toxic effect of acetylated tau on CMA described in this study could be responsible for the CMA dysfunctions observed in brains from AD patients and contribute to disease propagation, as demonstrated here in a mouse model of tauopathy. These findings on the mechanisms underlying autophagic clearance and cell-to-cell propagation of tau could guide future therapeutic interventions in tauopathies.

## Results

### Acetylated tau is degraded by macroautophagy

**Fig. 1:**
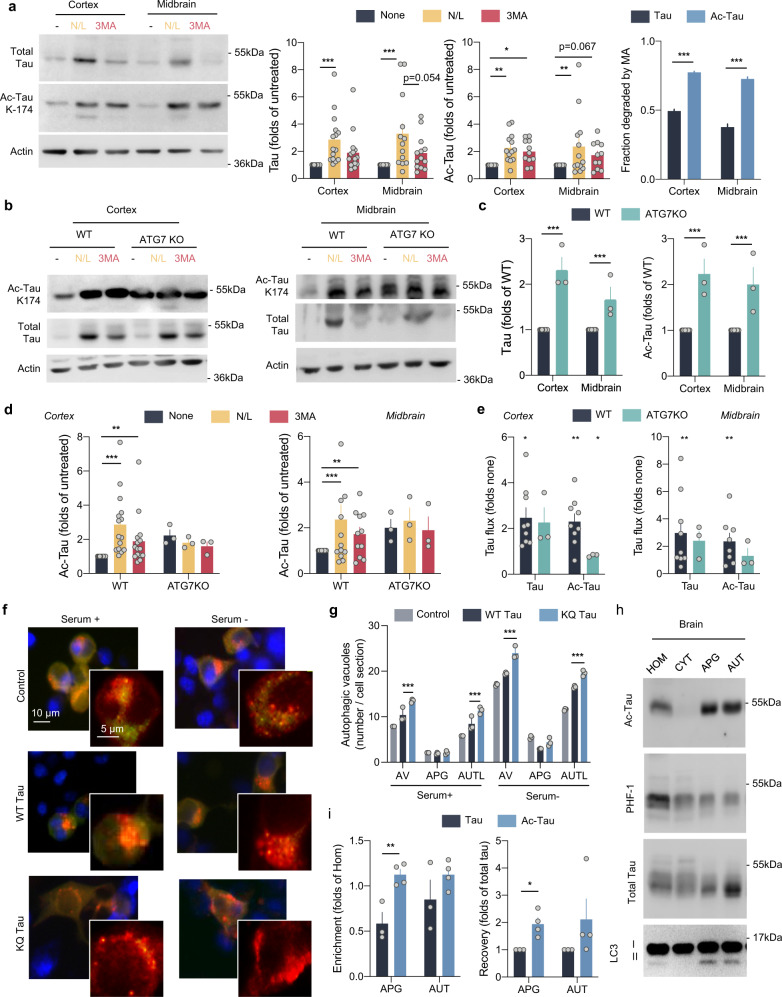
Acetylated tau is degraded by macroautophagy. **a** Brain slices from the mouse cortex or midbrain were incubated in the presence or absence of NH_4_Cl 20 mM and leupeptin 200 µM (N/L) or 3-methyladenine (3MA) 10 mM for 4 h at 37 °C and blotted for the indicated proteins. Right: Quantification of total tau (left) [Degradation pathway effect: *F*_(2,78) _= 14.31, *p* < 0.0001], acetylated tau (Ac-Tau; middle) [Degradation pathway effect: *F*_(2,78) _= 12.22, *p* < 0.0001], and fraction of tau degraded by macroautophagy (MA) [Tau status effect: *F*_(1,52) _= 507.2, *p* < 0.0001], *n* = 15 (for cortex) and 13 (for midbrain) mice per genotype. Controls of the selectivity of the acetylated antibody using brains from tau knockout mice and of the efficiency of the inhibitors are shown in Supplemental Fig. 1a, b. **b**–**e** Brain slices from wild-type (WT) and Atg7 knockout mice (ATG7KO) cortex or midbrain were incubated as in **a** and blotted for the indicated proteins. **b** Representative immunoblots. **c** Quantification of total and acetylated tau in two brain regions (cortex and midbrain) [Genotype effect: *F*_(1,28) _= 174, *p* < 0.0001]. **d** Changes of acetylated tau levels in both mice groups in the cortex (left) [Combined effect: *F*_(2,42) _= 4.280, *p* = 0.0203] and midbrain (right) [Degradation pathway effect: *F*_(2,42) _= 3.707, *p* = 0.0329], *n* = 3. Changes in total tau are shown in Supplemental Fig. 1d. **e** Flux of total and acetylated tau in the cortex (left) and midbrain (right) calculated as the fold increase in tau levels upon addition of N/L. **f** High content microscopy representative images of a mouse neuroblastoma cell line Neuro-2a (N2a) transduced with mCherry-GFP-LC3 and transfected with unmodified FLAG-wild-type Tau (WT) or acetylation-mimetic FLAG-K^274,281^Q tau (KQ) and maintained in the presence or absence of serum for 4 h. Nuclei are highlighted with DAPI in blue. Insets show higher magnification images of the two channels. *n* > 800 cells/condition in three different wells per day from cells transfected in 4 different days. **g** Quantification of total autophagic vacuoles (AV), autophagosomes (APG, yellow puncta), and autolysosomes (AUTL, red only puncta). *n* > 800 cells/condition in three different wells per day from cells transfected in 4 different days. Expression levels of both proteins upon transfection in N2a cells are shown in Supplemental Fig. 1h. [Serum+: Tau status effect: *F*_(2,18) _= 49.37, *p* < 0.0001; Serum−: Tau status effect: *F*_(2,18) _= 11.26, *p* = 0.0007] (**h**) Homogenate (HOM), Cytosol (CYT), Autophagosomes (APG), and Autolysosomes (AUT) were isolated from mouse brain and blotted for the indicated proteins. Representative immunoblot. *n* = 3 mice. **i** Quantification of enrichment (left) [Tau status effect: *F*_(1,10) _= 16.90, *p* = 0.0021] and recovery (right) [Tau status effect: *F*_(1,10) _= 16.92, *p* = 0.0021] of total tau and acetylated tau in the indicated fractions, *n* = 3 mice. All values are mean ± s.e.m. Statistical analysis were done using Two-way ANOVAs (panels **a**, **c**, **d**, **g**, and **i**) and two-tailed *t*-tests or Wilcoxon tests (panels **e**). Differences were significant with untreated or WT tau for *p < 0.05, **p < 0.01, ***p < 0.005. For clarity purposes, only relevant statistical comparisons are presented. Uncropped blots are shown in Supplemental Fig. 7.

To analyze the impact of tau acetylation on its degradation by autophagy in vivo, we first measured changes in levels of total and acetylated tau in different mouse brain regions upon blockage of all types of lysosomal degradation with a combination of ammonium chloride and leupeptin. As previously described in cultured cells^32^, we found that part of cellular tau was degraded in lysosomes, but that inhibition of macroautophagy with the PI3Kinase inhibitor 3-methyladenine (3MA) only prevented degradation of a fraction (about 37–49% depending on brain region) of tau protein (Fig. 1a). In contrast, acetylated tau at K174, which also displayed lysosomal degradation, was mostly degraded via macroautophagy (up to 80% of lysosomal degradation was prevented by 3MA) (Fig. 1a). Controls for the selectivity of the acetylated antibody using brains from tau knockout mice and of the efficiency of the inhibitors are shown in Supplemental Fig. 1a, b. In further support of preferential degradation of acetylated tau by macroautophagy, analysis of brains from mice with compromised macroautophagy (ATG7 knockout) revealed a significant accumulation of acetylated tau (Fig. 1b, c). Using the same combination of inhibitors of lysosomal proteolysis as above, we confirmed that lysosomal degradation of acetylated tau was severely compromised in brains from ATG7KO mice when compared to wild type littermates (Fig. 1d, e), whereas lysosomal degradation of unmodified tau protein was still observed in macroautophagy-deficient brains (Fig. 1b, e and Supplemental Fig. 1d; note: the unexpected increase in total tau levels observed upon 3MA treatment in the cortex of ATG7KO mice cannot be due to changes in tau degradation by macroautophagy since this pathway is blocked in these animals, and it likely results from non-specific effects of PI3Kinase inhibition in these mice).

Differences in tau clearance can be directly attributed to the acetylation of the protein since we could reproduce them using flag-tagged wild-type (WT) and an acetylation-mimetic form of tau (mutation of lysine 274 and 281 to glutamine; KQ tau) expressed at comparable levels in mouse embryonic fibroblasts (MEFs) (Supplemental Fig. 1e). As in the mouse brains, WT tau was degraded primarily in lysosomes through a pathway insensitive to 3MA, but the degradation of the acetylation-mimetic form of tau occurred via macroautophagy and to a lesser extent the proteasome (as reflected by sensitivity to the proteasome inhibitor) (Supplemental Fig. 1f; efficiency of the inhibitors is shown in Supplemental Fig. 1g).

Two additional pieces of evidence supported the proposed preferential degradation of acetylated tau through macroautophagy. First, we observed an increase in macroautophagy activity in cultured cells upon expression of the acetylation-mimetic form of tau (Supplemental Fig. 1h). Macroautophagy was monitored by transducing cells with the tandem reporter mCherry-GFP-LC3^44^ to quantify the number of autophagosomes (puncta positive for both fluorophores) and maturation of autophagosomes into autolysosomes (mCherry-only positive puncta due to quenching of GFP fluorescence at the low pH upon fusion with lysosomes). We found that expression of KQ tau led to a significant increase in basal and in serum-removal induced macroautophagic flux compared to WT tau (Fig. 1f, g). This increase in macroautophagy in response to elevated levels of acetylated tau could be elicited to facilitate its elimination through this pathway. In agreement with this hypothesis, analysis of the forms of tau detected in autophagosomes and autolysosomes isolated from wild-type mice brains revealed significantly higher enrichment of acetylated tau in these fractions (folds increase of acetylated tau per microgram of protein in the fractions relative to the homogenate) when compared with total or phosphorylated forms of tau (Fig. 1h, i and Supplemental Fig. 1i). In fact, the percentage of cellular acetylated tau recovered in autophagosomes (recovery) at a given time is almost double that of total tau (Fig. 1h, i), further supporting that acetylated tau is actively targeted to these autophagic compartments. Overall, these findings support that acetylated forms of tau are preferentially degraded in the lysosomal system via macroautophagy.

### Reduced CMA contributes to the accumulation of acetylated tau

**Fig. 2:**
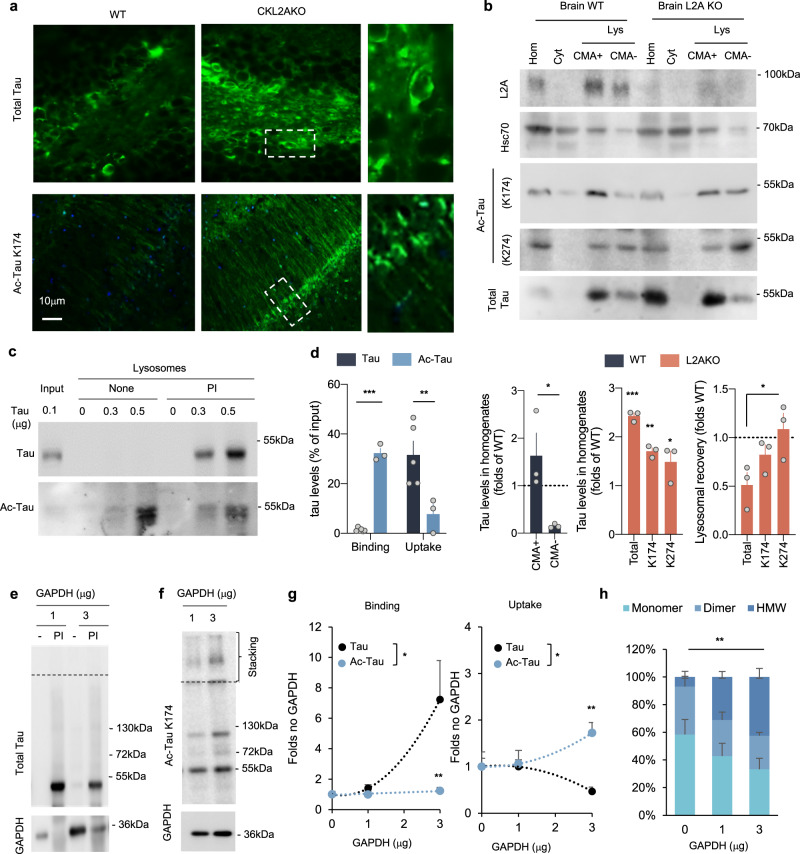
Acetylated tau associates with CMA-active lysosomes. **a** Immunofluorescence staining for tau and acetylated tau (Ac-Tau) of brain sections from wild-type (WT) and CaMKinaseIIα-Cre-L2A knockout mice (L2AKO). *n* = 3 mice. **b** Immunoblot for the indicated proteins of homogenate (Hom), Cytosol (Cyt), and two lysosomal (Lys) populations with different CMA activity (CMA+ and CMA−) isolated from WT and L2AKO mice brains. Bottom left plot: Quantification of the percentage of total tau recovered in the indicated fractions. [Two-tailed *t*-test *t*_4_ = 3.079, *p* = 0.037]. Bottom middle plot: Quantification of tau levels in L2AKO mice brain homogenate relative to WT (dotted line) [Two-way ANOVA Tau status effect: *F*_(1,12) _= 90.17, *p* < 0.0001]. Bottom, right plot: Quantification of recovery of tau levels in lysosomes from L2AKO mice brain relative to WT [One-way ANOVA interaction effect: *F*_(2,6) _= 4.58, *p* = 0.061; Total vs. K274 *p* = 0.041] *n* = 3 mice. **c** Immunoblots of isolated lysosomes pretreated or not with protease inhibitors (PI) and incubated with increasing concentrations of tau untreated (Tau) or acetylated in vitro (Ac-Tau). **d** Binding and uptake of tau calculated from quantification of immunoblots as the ones shown in **c** [Two-way ANOVA interaction effect: *F*_(1,13) _= 49.67, *p* < 0.0001], *n* = 3 independent experiments. **e**, **f** Effect of increasing concentrations of glyceraldehyde 3-phoshpate dehydrogenase (GAPDH) in the association of tau (**e**) and Ac-Tau (**f**) with isolated liver lysosomes pre-incubated or not with protease inhibitors (PI) as labeled. The dashed line indicates the separation between running and stacking. *n* = 3 independent experiments per type of tau and amount of GAPDH. **g** Quantification of the effect of increasing concentrations of GAPDH on binding [Two way ANOVA Tau status effect: *F*_(1,12) _= 6.033, *p* = 0.0302] and uptake [Two-way ANOVA Tau status effect: *F*_(1,12) _= 5.815, *p* = 0.0328] of control and acetylated tau (note that acetylated includes all variants independently of their molecular weight). *n* = 3 independent experiments. **h** Quantification of the percentage of lysosome-associated acetylated tau detected as monomer, dimer, or higher molecular weight (HMW) complexes in the stacking of the gel upon addition of GAPDH. *n* = 3 independent experiments [Two-way ANOVA interaction effect: *F*_(4,18) _= 4.894, *p* = 0.0076]. All values are mean ± s.e.m. Differences with unmodified tau were significant for *p < 0.05 05, **p < 0.01, ***p < 0.005. Uncropped blots are shown in Supplemental Fig. 7.

Although most acetylated tau was undergoing degradation via macroautophagy, analysis of brains from mice with specific CMA blockage in pyramidal neurons (CKL2AKO) (Supplemental Fig. 2a) revealed an increase in both unmodified and acetylated tau (Fig. 2a; controls are shown in Supplemental Fig. 2b). Since we have previously shown that macroautophagy is fully functional in CKL2AKO mice, it was more likely that the accumulation of acetylated tau was related to changes in tau itself, which motivated us to analyze the interplay of acetylated tau and CMA in more detail.

Using mouse brains to isolate subpopulations of lysosomes with different CMA activity^45^ (organelle markers are shown in Supplemental Fig. 2c), we confirmed in vivo our earlier findings that a fraction of cellular tau undergoes degradation via CMA^29,32^ and consequently can be detected preferentially in the lysosomes with higher activity for this pathway (higher content of hsc70 in their lumen) (Fig. 2b, bottom left graph). In contrast, the amount of tau in lysosomes isolated from L2AKO brains was markedly reduced (Fig. 2b, note that although the abundance of tau in L2AKO brains was double than in wild-type littermates (bottom middle graph), the amount of total tau recovered in lysosomes from L2AKO mice was almost half than that in lysosomes from wild-type mice (bottom right graph)). The observed changes in lysosomal tau were not due to changes in the overall abundance of lysosomes in L2AKO mice, which were comparable in number to those in wild-type mice (Supplemental Fig. 2d). Using antibodies specific for tau acetylated on different residues, we found that some of the acetylated variants (i.e., acetylation on K174) were detected in more abundance in CMA-active lysosomes, but that in contrast with total tau, blockage of CMA did not reduce their levels in these lysosomes (Fig. 2b, bottom right graph), in support of tau association with the lysosomal surface without internalization. In agreement with our previously detected degradation of acetylated tau by macroautophagy (Fig. 1), other acetylated forms of tau (i.e., acetylation on K274) were detected in similar amounts within CMA lysosomes and the group of lysosomes usually engaged in macroautophagy (CMA-)^46^ (Fig. 2b).

The increase in total levels of acetylated tau upon CMA blockage in vivo and the preferential association of some acetylated forms of tau with lysosomes active for CMA led us to directly analyze the translocation of acetylated tau inside lysosomes via CMA. We acetylated purified tau (multi-acetylation was confirmed with antibodies specific for different tau acetylation sites, Supplemental Fig. 2e) and presented both acetylated and non-acetylated tau to isolated intact lysosomes in a well-established in vitro system that allows recapitulating binding and translocation of CMA substrates inside lysosomes^47^. Binding is quantified as the amount of tau associated with lysosomes at the end of the incubation (because the one internalized is rapidly degraded), and uptake is the difference between binding and the amount of tau associated with lysosomes pretreated with protease inhibitors (to prevent degradation of the internalized tau). Unmodified tau was rapidly internalized and degraded in lysosomes (as it could only be detected in lysosomes pre-treated with protease inhibitors), whereas acetylated tau remained for the most part associated with the membrane and failed to translocate (Fig. 2c, d). We confirmed the absence of any proteasome contamination in the isolated lysosomes that could account for tau degradation (Supplemental Fig. 2f), and demonstrated that uptake of unmodified tau was through CMA as it could be outcompeted by the addition of GAPDH, a well-known CMA substrate^48^ (Fig. 2e, g). In contrast, the addition of GAPDH did not reduce the amount of acetylated tau associated with lysosomes, and instead, we observed the gradual formation of a high molecular weight complex of acetylated tau with some of them remaining in the stacking of the gel (Fig. 2f–h). These results support that acetylation of tau markedly reduces its degradation through CMA and that the reduced dynamics of translocation may facilitate the irreversible formation of larger molecular weight complexes of acetylated tau at the lysosomal membrane, which could further compromise substrate uptake in these organelles.

### Acetylated tau reduces CMA activity

**Fig. 3:**
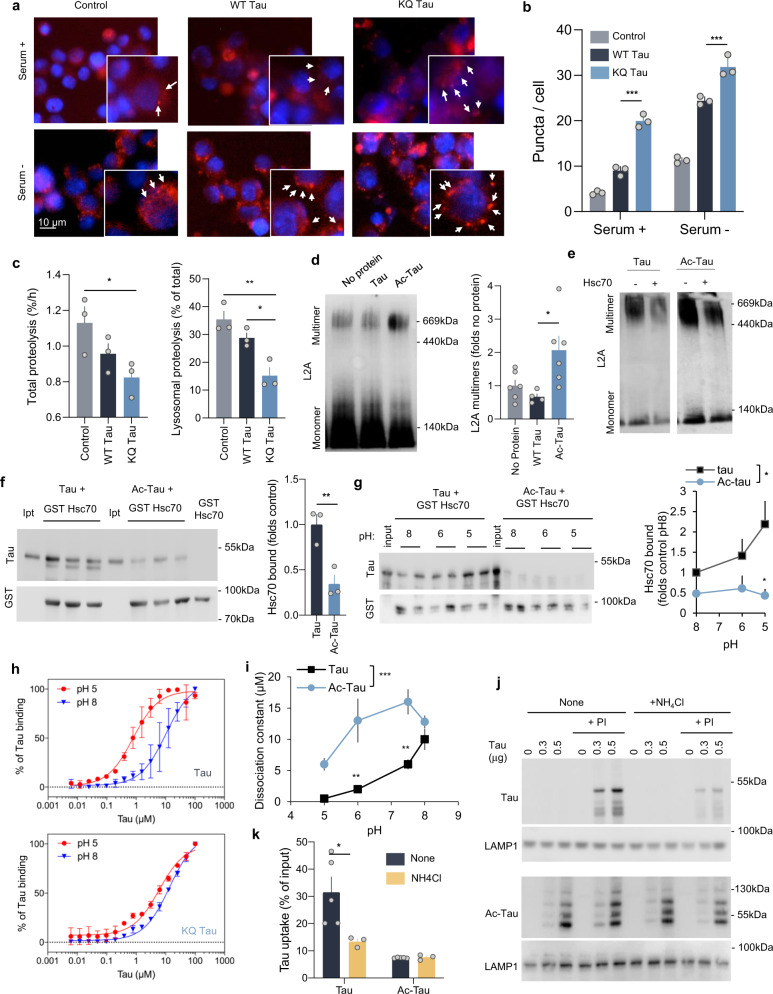
Acetylated tau has an inhibitory effect on CMA activity. **a** Representative images of mouse neuroblastoma cell line Neuro-2a (N2a) transduced with KFERQ-PS-Dendra2 and transfected with empty vector (Control), FLAG-wild-type tau (WT Tau) or FLAG-K^274,281^Q tau (KQ Tau), and maintained in the presence (+) or absence (−) of serum for 16 h. Nuclei are highlighted with DAPI in blue. Insets show higher magnification images and arrows point examples of fluorescent puncta. *n* > 800 cells/condition in three different wells and 4 independent experiments. **b** Quantification of an average number of puncta per cell section. [Two-way ANOVA Tau status effect: F_(2,12) _= 179.1, *p* < 0.0001], *n* > 800 cells/condition in three different wells, and *n* = 4 independent experiments. Controls for tau expression are shown in Supplemental Fig. 1h (protein levels) and Supplemental Fig. 3a (mRNA levels). **c** Degradation rates of long-lived proteins in N2a cells transfected as in **a** and radiolabeled for 48 h with ^3^H-leucine), [One-way ANOVA Tau status effect: *F*_(2,6) _= 4;619, *p* = 0.0610]. Right: fraction of degradation occurring in lysosomes (sensitive to inhibition of lysosomal proteolysis), [One-way ANOVA Tau status effect: *F*_(2,6) _= 14.55, *p* = 0.005], *n* = 3 independent experiments. **d** Immunoblot for L2A of isolated lysosomes subjected to blue-native electrophoresis after being incubated alone (No protein) or with unmodified (Tau) or in vitro acetylated tau (Ac-Tau). A 700-kDa multimeric complex and the 110-kDa monomer are shown. Right: Quantification of multimeric levels of L2A relative to those in lysosomes incubated alone. [One-way ANOVA Tau status effect: *F*_(2,13) _= 5.580, *p* = 0.0178], *n* = 4 independent experiments. **e** Lysosomes incubated and processed under the same conditions as in **d** but in the presence or absence of hsc70. **f** Immunoblot for tau of the GST-pulldowns of GST-hsc70 incubated with tau or Ac-Tau at neutral pH for 30 min at 37 °C. Input (Ipt): 1/10 of tau added to the incubation. Right: Quantification of tau bound to Hsc70. [Two-tailed *t*-test *t*_4_ = 4.432, *p* = 0.0114], *n* = 3. **g** Samples incubated as in **f** but at different pH. Right: Quantification of tau bound to hsc70 [Two-way ANOVA Tau status effect: *F*_(1,18) _= 10.85, *p* = 0.0040], *n* = 4 independent experiments. **h** Tau or K^280^Q tau binding assay to immobilized hsc70 determined by ELISA at the indicated pH. See also Supplemental Fig. 3b and c for binding assays at intermediate pH. *n* = 6 (in 2 independent experiments). **i** Summary plot of the binding assay at all tested pH for tau and acetylated tau (Ac-Tau). [Two-way ANOVA Tau status effect: *F*_(1,8) _= 42.10, *p* = 0.0002]. *n* = 6 (in 2 independent experiments). **j** Immunoblots of lysosomes from starved rat livers, pretreated or not with protease inhibitors (PI) and/or NH_4_Cl 20 mM for 10 min at 4 °C and then incubated with tau (left) or Ac-Tau (middle). *n* = 3 independent experiments. **k** Quantification of tau proteins binding and uptake by lysosomes. [Two-way ANOVA Tau status effect: *F*_(1,17) _= 17.22, *p* = 0.0013], *n* = 3 independent experiments. All values are mean ± s.e.m. Differences with control or unmodified tau were significant for *p < 0.05, **p < 0.01, ***p < 0.005. Uncropped blots are shown in Supplemental Fig. 7.

We have recently shown that pathogenic tau mutants that fail to undergo degradation by CMA persist associated with the lysosomal membrane and interfere with the degradation of other proteins through this pathway^32^. To determine if the acetylated tau that we found persistently associated with the lysosomal membrane could also be toxic for CMA, we analyzed CMA activity in cells expressing WT or the acetylation-mimetic form of tau (control of tau expression is shown in Supplemental Fig. 3a). We monitored CMA in cultured cells using photoswitchable Dendra fluorescent protein-tagged with the CMA-targeting motif (KFERQ-PS-Dendra^49^) that upon CMA activation relocalizes from the cytosol to the surface of lysosomes giving a punctate fluorescent pattern. Transfection of neuroblastoma cells stably expressing the CMA reporter with WT tau increased the number of fluorescent puncta and this increase was significantly more accentuated in cells expressing KQ tau (Fig. 3a, b). KFERQ-PS-Dendra protein only preserves its fluorescence while bound to the cytosolic side of the lysosomal membrane but stops fluorescing once internalized due to its unfolding during translocation^49^. Consequently, to validate whether the augmented association of CMA substrates with lysosomes in cells expressing the tau proteins resulted in increased translocation and degradation, we measured the rates of degradation of long-lived proteins, typical autophagy substrates, under the same conditions. Contrary to the CMA reporter data, we found that total protein degradation rates were significantly reduced upon expression of the acetylation-mimetic form of tau and that this decrease was even more pronounced when only the 3MA-insensitive lysosomal proteolysis (no macroautophagy) was accounted for (Fig. 3c). These results suggest that an increase in the cellular levels of acetylated tau leads to reduced rates of CMA and that this blockage does not interfere with substrate binding with the lysosomal surface, but it mainly affects substrate translocation inside lysosomes.

To further elucidate the mechanism behind the observed CMA blockage by acetylated tau, we analyzed the lysosomal translocation complex that forms by oligomerization of L2A^41^ and can be detected as a 700 kDa complex after subjecting lysosomes to blue-native electrophoresis and immunoblot for L2A^50^. This complex is rapidly disassembled into L2A monomers as soon as translocation is completed to allow for a new cycle of binding/translocation^41^. We found a significant increase in the amount of L2A organized into translocation complexes upon exposure of lysosomes to acetylated tau that was not detected when using unmodified tau (Fig. 3d and Supplemental Fig. 3b). We considered the possibility that acetylated tau primarily inhibited L2A disassembly leading to an abnormally higher number of translocation complexes. However, disassembly of L2A in the presence of acetylated tau could still be forced by adding an excess of cytosolic hsc70 (previously shown to contribute to the dissociation of L2A from the multimeric complex^41^) (Fig. 3e) or with chemical activators of hsc70^51^ (Supplemental Fig. 3b).

Next, we investigated possible changes in the other components that contribute to substrate translocation, namely hsc70 present at the cytosolic and in the luminal side of the lysosomal membrane^39,42,45,52^. Incubation of the tau proteins with recombinant GST-hsc70 revealed lower binding of acetylated tau to hsc70 as compared to unmodified tau (Fig. 3f). Because the luminal lysosomal resident hsc70 completes translocation of substrates by binding to them as they enter the lysosomal acidic environment^42^, we tested the effect of acidification on the tau/hsc70 binding and found a very pronounced increase in binding between both proteins as pH decreases (Fig. 3g). These pH-dependent differences in affinity for hsc70 provide a possible explanation for the unusual rapid translocation of tau into lysosomes when compared to other CMA substrates. Interestingly, binding between hsc70 and acetylated tau did not increase at lower pH, and instead, acidification seemed to further reduce both proteins’ interaction (Fig. 3g). We confirmed the pH dependence of hsc70 and tau binding, with tighter binding at pH5 than pH8, using a quantitative ELISA-based binding assay with immobilized hsc70 (Fig. 3h, top and Supplemental Fig. 3c). Comparison of binding of unmodified and KQ tau in the same assay revealed lower binding affinity of hsc70 for the acetylation-mimetic tau protein more evident at low pH (dissociation constants at pH 5 of 0.78 and 5.91 for WT and KQ tau, respectively) (Fig. 3h, i and Supplemental Fig. 3d). In fact, when comparing the binding of KQ tau to hsc70 at different pH, the enhanced binding at low pH, observed for control tau, was no longer evident for the modified tau protein (Fig. 3h, bottom).

To functionally validate that stronger hsc70/tau binding at low pH was required for efficient lysosomal translocation of tau, we analyzed the effect of dissipating lysosomal pH on tau translocation. Neutralization of the lysosomal pH by incubation with ammonium chloride did not affect lysosomal membrane stability (Supplemental Fig. 3e) or the ability to translocate other CMA substrates into lysosomes (GAPDH shown in Supplemental Fig. 3f). However, translocation of tau was markedly reduced when the pH gradient across the lysosomal membrane was no longer present, further supporting that the unusually fast translocation of tau into lysosomes is dependent on its higher binding affinity with hsc70 at low pH (Fig. 3j,k). Neutralization of the lysosomal pH did not significantly affect the already low lysosomal translocation of acetylated tau (Fig. 3j,k). Overall, these results suggest that the fast translocation of tau into lysosomes via CMA relies heavily on the pH-sensitivity of hsc70/tau binding and that acetylation of tau reduces the efficiency of its lysosomal translocation, at least in part, because of the loss of this pH sensitivity for hsc70 binding.

### Tau rerouting to e-MI contributes to its extracellular release

**Fig. 4:**
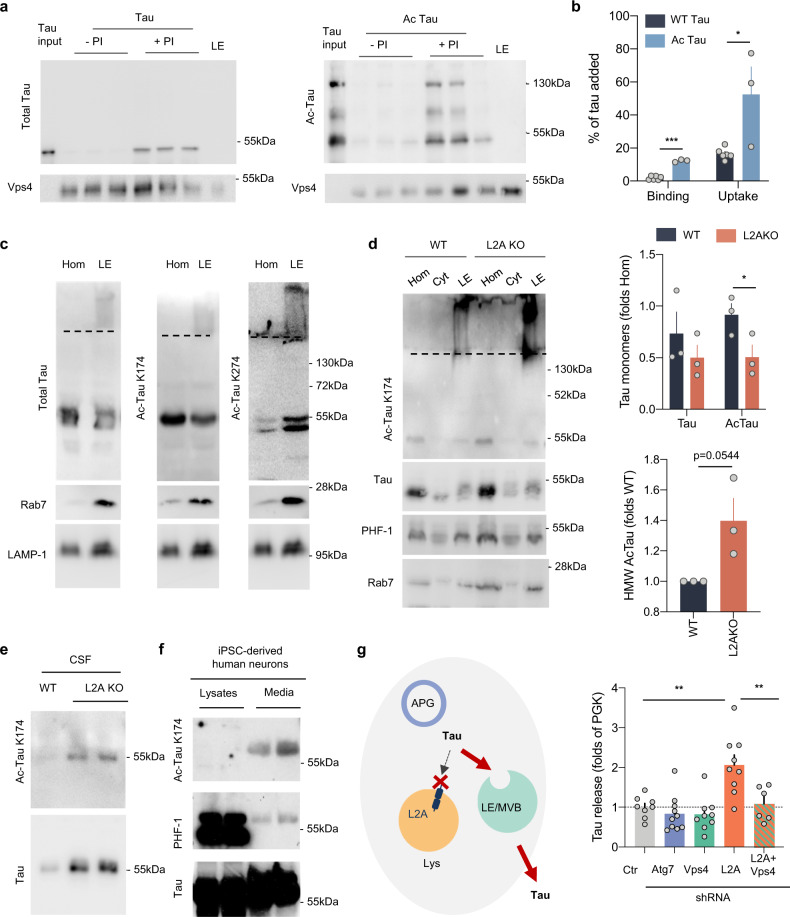
Acetylated tau and endosomal microautophagy. **a** Immunoblots of late endosomes (LE) isolated from tau-null mice brains, pretreated or not with protease inhibitors (PI), and incubated with tau or in vitro acetylated tau (Ac-tau). Tau input is 1/5 of the protein added. **b** Quantification of endosomal binding [Two-tailed *t*-test *t*_7_ = 13.80, *p* < 0.0001] and uptake [Two-tailed *t*-test *t*_7_ = 3.239, *p* = 0.0143] of tau proteins from blots as in **a**. *n* = 3 mice. **c** Immunoblot with the indicated antibodies of homogenate (Hom) and late endosomes (LE) isolated from mice brain. A dashed line indicates the separation between running and stacking in the gel. The same homogenate and LE fractions were loaded in triplicate in a membrane and strips of the membrane were separately incubated with each of the antibodies to allow for comparison among antibodies. **d** Immunoblot for the indicated antibodies of Hom, cytosol (Cyt), and LE isolated from brains of wild-type (WT) and L2A knock-out mice (L2A KO). Dashed lines as in **c**. Right: quantification of the amount of total and acetylated tau in monomer [Two-way ANOVA genotype effect: *F*_(1,8) _= 10.56, *p* = 0.0117] and in high molecular weight (HMW) complexes [Two-tailed *t*-test *t*_4_ = 2.694, *p* = 0.0545]. *n* = 4 mice. **e** Immunoblot for total and acetylated tau in CSF isolated from wild-type and L2AKO mice. *n* = 3 mice per genotype were pooled for each immunoblot; the 2 samples shown here correspond to isolations done in 2 separate days. **f** Immunoblot for the indicated forms of tau of lysates and culture media from iPSC-derived human neurons upon stimulation with KCl for 30 min. Note: saturated bands are shown for the lysates in the PHF-1 membrane to illustrate the small amount of phosphorylated tau detected in the media. *n* = 4 different experiments performed in 2 different iPSC cell lines. **g** Left: Scheme illustrating rerouting of tau towards LE/MVB upon blockage of CMA and extracellular release of tau after fusion of LE/MVB with the plasma membrane. APG: autophagosome, Lys: lysosome. Right: Quantification of extracellular tau using ELISA upon blockage of the various autophagy pathways in N2a cells expressing human ^P301L^Tau. Vps4: VPS4 knock-down, L2A: L2A knock-down, Atg7: ATG7 knock-down, L2A + Vps4: a combination of L2A and VPS4 knock-down. The efficiency of the different knock-downs is shown in Supplemental Fig. 4c. Values are expressed as the fold on PGK (Control). [One-way ANOVA *F*_(__4,37)_ = 9.542, *p* < 0.0001]. *n* = 8, 10, 9, 9, 6 (in order of genotype shown in the graph) independent experiments. All values are mean ± s.e.m. Differences with control or unmodified tau were significant for *p < 0.05, **p < 0.005. Uncropped blots are shown in Supplemental Fig. 7.

Hsc70 also mediates selective degradation of cytosolic proteins in late endosomes through endosomal microautophagy (e-MI)^43^. In light of the negative impact of tau acetylation on its degradation by CMA and our recent findings that a fraction of unmodified tau undergoes e-MI degradation^32^, we investigated whether acetylation affected the degradation of tau through e-MI. We first presented the purified tau proteins to isolated late endosomes/multivesicular bodies (LE/MVB) from tau-null mice brains incubated or not with protease inhibitors to assess the association and degradation of exogenously added tau in these compartments. We found that unmodified tau was binding to and taken up by LE/MVB and that this process was even more efficient for acetylated tau (Fig. 4a, b). Interestingly, in the case of acetylated tau, not only the monomer but also high molecular weight forms of the protein could be detected in LE/MVB but only when proteolysis was prevented (Fig. 4a, b), in agreement with the previously described ability of e-MI to internalize and degrade fully folded proteins even when organized into oligomeric complexes^53^.

Immunoblot of mouse brain LE/MVB for tau confirmed that a fraction of the endogenous protein was detectable in these compartments both as monomer and as high molecular weight protein complexes retained for the most part in the stacking of the gel (Fig. 4c). Using the antibodies specific for acetylation of tau at different lysine residues, we found that some of the acetylation variants, but not phosphorylated tau, were highly enriched in LE/MVB either as monomers and/or as high molecular weight protein complexes (Fig. 4c and Supplemental Fig. 4a). These results suggest that even in the brains of young healthy mice, a fraction of acetylated tau organizes into irreversible high molecular weight protein complexes that seem to be cleared from the cytosol via e-MI. Interestingly, the amount of high molecular weight forms of acetylated tau reaching LE/MVB markedly increased in the brains of mice with compromised CMA (L2AKO mice) (Fig. 4d) making us hypothesize that e-MI could be a rerouting mechanism for the acetylated tau that oligomerized upon CMA blockage.

To gain a better understanding of the consequences of tau rerouting to LE/MVB and to confirm that delivery of tau to LE used conventional e-MI mechanisms, we knocked down Vps4—one of the components of the endosomal sorting complexes required for transport previously shown necessary for e-MI^43^—in cells expressing tau. Knock-down of Vps4 led to increased cellular levels of tau, reduced tau degradation, and resulted in a decrease in the discrete amount of tau detected in the extracellular media in these cells (Supplemental Fig. 4b). Since LE/MVB can fuse with the plasma membrane and release their content extracellularly^54,55^, we contemplated the possibility that the presence of tau in LE/MVB could be a route for extracellular release. In fact, we noticed that CSF from L2AKO mice contained a higher abundance of tau compared to wild-type littermates and that a large fraction of this extracellular tau was acetylated (Fig. 4e), coinciding with our observation of increased content of acetylated tau in LE/MVB from these mice (Fig. 4d). We found that acetylated tau was also the form of the protein preferentially detected in the extracellular media of iPSC-derived human neurons upon induction of its release as previously described by neuronal activation^21^ (Fig. 4f).

To directly test the contribution of changes in the degradation pathway of tau to its extracellular release, we next used ELISA to measure extracellular tau upon blockage of the different autophagy pathways in N2a cells (Fig. 4g; Supplemental Fig. 4c). Contrary to recent reports in other cell types supporting that ESCRT machinery is required for macroautophagy^56^, knock-down of Vps4 in N2a cells did not reduce macroautophagy activity in these cells (Supplemental Fig. 4d, e). In fact, upon microautophagy blockage, we observed a consistent increase in macroautophagy flux and autolysosome number (Supplemental Fig. 4d), likely of compensatory nature, since, despite increased rates of macroautophagy-dependent degradation in Vps4 knock-down cells, overall proteolysis remained unchanged from control cells (Supplemental Fig. 4e). The presence of fully functional macroautophagy upon Vps4 knock-down made this intervention suitable to assess the contribution of e-MI to tau secretion. Blockage of e-MI (Vps4 knock-down) or macroautophagy (Atg7 knock-down) alone resulted in a modest but consistent decrease in extracellular tau, whereas blockage of CMA led to a massive increase in extracellular tau release (Fig. 4g), consistent with the higher abundance of tau in CSF from L2AKO mice (Fig. 4e). Interestingly, the release of extracellular tau upon CMA blockage was completely prevented if we imposed e-MI blockage on CMA-deficient cells (Fig. 4g). These findings are in agreement with our proposed model of active rerouting of tau to e-MI upon CMA blockage and its extracellular release from LE/MVB (Fig. 4g).

Altogether, these results show that e-MI also contributes to the sequestration of acetylated tau, especially in the context of CMA deficiency, and that the presence of tau in LE/MVB provides a route for its active extracellular release.

### CMA dynamics are altered in AD patient brains

**Fig. 5:**
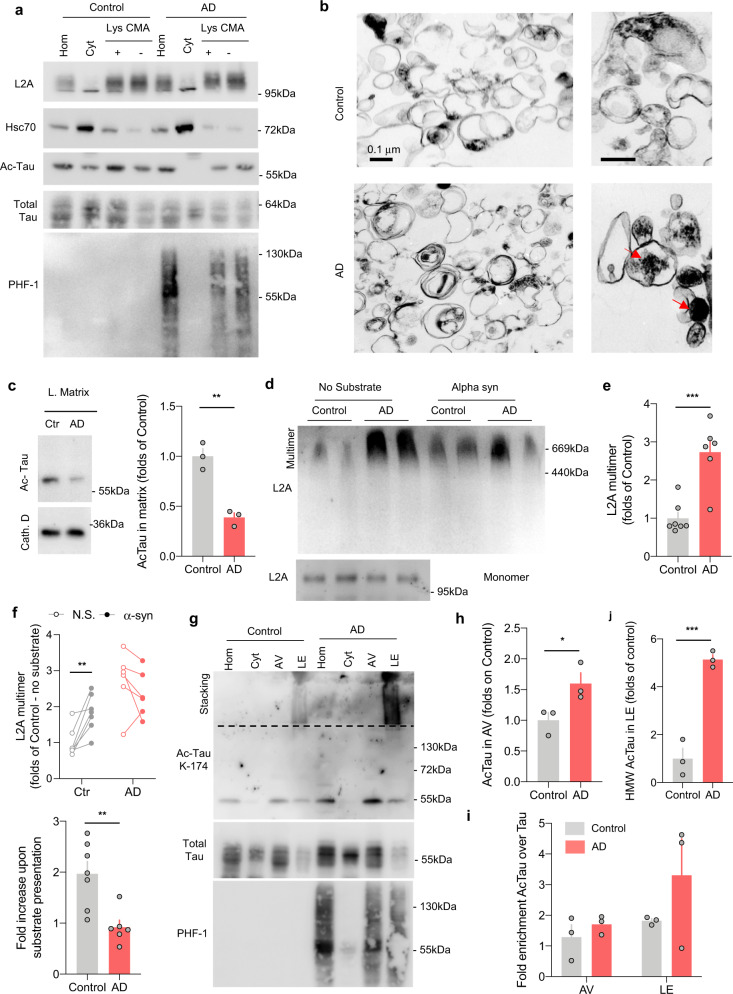
Changes in the association of tau with autophagic and lysosomal compartments in Alzheimer’s disease patient brains. **a** Immunoblots for the indicated proteins of homogenates (Hom), cytosol (Cyt), and two populations of lysosomes (Lys) with different CMA activity (CMA+ and CMA−) isolated from the frontal area of brains from patients with Alzheimer’s Disease (AD, *n* = 6) and non-neurological patients of matching age (Control, *n* = 7). **b** Pictures of CMA-active lysosomes isolated from control (*n* = 3) or AD (*n* = 3) patients’ brains and subjected to electron microscopy. Right: higher magnification inserts. Red arrows: undegraded dense lipofuscin-like material. Differences noted in the images were reproducible in the 3 brains. **c** Immunoblot for acetylated tau (Ac-Tau) in lysosomal matrices from control and AD patients. Right: Quantification of the amount of acetylated tau (Ac-Tau) internalized in AD patients’ lysosomes relative to control [Two-tailed *t*-test *t*_4_ = 6.831, *p* = 0.0024]. *n* = 3 samples (1 per brain) per group. **d** Immunoblot from L2A of the blue-native electrophoresis of lysosomes isolated as in **a** and incubated alone (No substrate) or in the presence of 1 µg of purified alpha-synuclein (Alpha syn). Two different individuals per diagnosis are shown. Multimer: indicates the 700-kDa multimeric translocation complex. Monomer: insert of the monomer run in a different gel. *n* = 6 control and 6 AD patient brains. **e** Quantification of multimeric L2A levels relative to those in control lysosomes in absence of substrate [Two-tailed *t*-test *t*_11_ = 4.936, *p* = 0.0004]. *n* = 7 control and 6 AD patient brains. **f** Quantification of multimeric L2A levels in absence of substrate (N.S.) or upon substrate (α-syn) presentation. Top changes of multimeric L2A for individual subjects. Bottom: Fold increase of L2A multimers upon substrate presentation [Two-tailed *t*-test t_11_ = 3.607, *p* = 0.0041]. *n* = 7 controls and 6 AD patient brains. **g** Immunoblots for the indicated proteins of homogenates (Hom), cytosol (Cyt), autophagic vacuoles (AV), and late endosomes (LE) isolated from patients with Alzheimer’s Disease (AD) and non-neurological patients of matching age (Control). *n* = 3 samples (1 per brain) per condition. **h** Quantification of the abundance of acetylated tau in autophagic vesicles [Two-tailed *t*-test *t*_4_ = 2.805, *p* = 0.0486]. *n* = 3 samples (1 per brain) per group. **I**, **j** Quantification of the ratio of acetylated to total tau in the indicated fraction (**i**) and of the abundance of the high molecular weight (HMW) acetylated tau species in LE (**j**). [Two-tailed *t*-test *t*_4_ = 8.719, *p* = 0.001]. *n* = 3 samples (1 per brain) per group. All values are mean ± s.e.m. Differences with control were significant for *p < 0.05, **p < 0.01, ***p < 0.005. Uncropped blots are shown in Supplemental Fig. 7.

Levels of acetylated tau are elevated in post-mortem brain samples from patients with various tauopathies^7,8^. To determine whether the changes imposed by acetylated tau on autophagy in the in vitro systems—namely, reduced CMA through disruption of lysosomal L2A dynamics and rerouting of acetylated tau oligomers to e-MI—were also observed in the brains of patients with Alzheimer’s disease (AD), we isolated two populations of lysosomes with different CMA activity, LE/MVB and autophagosomes from brains of 6 patients with autopsy-confirmed AD and 7 age-matched autopsy confirmed controls (no-AD) (patient information is summarized in “Methods” section). AD diagnosis was also supported by the marked differences in levels of hyperphosphorylated tau between control and AD brains (Supplemental Fig. 5a). Analysis of the different autophagic compartments (Supplemental Fig. 5b–d show enrichment of relevant organelle markers) demonstrated acetylated tau associated with lysosomes isolated from control individuals and, similar to mouse brain lysosomes, a preferential enrichment of this form of the protein in the group of lysosomes active for CMA (Fig. 5a). Interestingly, the pattern of lysosomal association of acetylated tau in the human AD brains (Fig. 5a) remarkably resembled the one observed in the brains of mice with compromised CMA (Fig. 2b), whereby, the preference for CMA-active lysosomes is lost and acetylated tau redistributed evenly between both subgroups of lysosomes, indirectly suggesting a possible compromise of CMA in these patients.

The reduced levels of luminal hsc70 in the population of CMA-active lysosomes in AD (usually related to loss of stability of this chaperone in the lysosomal lumen upon loss of lysosomal acidification^45^) (Fig. 5a), as well as the higher presence of undegraded cargo in lysosomes isolated from AD brains (Fig. 5b), is in agreement with the reported loss of lysosomal acidification in some types of AD^36,57^. We hypothesized that the possible loss of pH gradient in AD brain lysosomes, along with the presence of acetylated tau protein at the lysosomal membrane (Fig. 5c and Supplemental Fig. 5f, confirms a 50% reduction in the amount of acetylated tau that reaches the lysosomal lumen), could result in similar interruption of lysosomal translocation of tau proteins and persistence of L2A into multimeric complexes to the one observed in the in vitro system (Fig. 3d). In fact, we found that despite their similar levels of L2A (Fig. 5a), a significantly larger fraction of this protein remained organized into multimeric complexes in the human AD brain lysosomes (Fig. 5d, e). While lysosomes from control brains exposed to a CMA substrate (α-synuclein shown here) display an increase in the multimeric levels of L2A to facilitate substrate uptake, levels of multimeric L2A in AD patient lysosomes exposed to the CMA substrate fail to increase and even decrease in some AD brains (Fig. 5d, f), further supporting altered dynamics of L2A. Our findings support deficient functioning of CMA in AD patients due to the inability of L2A to go through normal rounds of assembly and disassembly.

Autophagic vacuoles isolated from AD brains (Fig. 5g, h, and Supplemental Fig. 5c) displayed higher content of acetylated tau (1.6 folds) than those from control brains, reflecting in part the overall cellular increase in acetylated tau and in agreement with the preferential degradation of this form of the protein by macroautophagy observed in vitro and in mouse brain sections (Fig. 1). Interestingly, while the ratio of acetylated to total tau in AD autophagic vacuoles was comparable between control and AD, in support of higher overall entrapment of tau by autophagosomes, we noticed a marked shift to an increase in the fraction of acetylated tau in the LE/MVB isolated from AD brains (Fig. 5g, i and Supplemental Fig. 5d). Almost all of the acetylated tau in AD LE/MVB was in the form of irreversible higher molecular weight protein complexes (Fig. 5g, j), strikingly resembling those observed in brains of CMA-incompetent mice (L2AKO) (Fig. 4d). Immunoblot with a specific antibody against oligomers of human tau confirmed the oligomeric nature of the protein trapped in LE/MVB and higher abundance of oligomers in LE/MVB from AD brains (Supplemental Fig. 5g). The LE/MVB-associated oligomeric complexes were in contrast barely detectable with the antibody against total tau, in further support of a preferential entrapment of only acetylated tau proteins (Fig. 5g and Supplemental Fig. 5e). These results demonstrate the conservation of the molecular signature of the autophagic defects, observed in vitro and in the experimental models, in the AD brain, and support the occurrence of acetylated tau rerouting to LE/MVB in AD patients’ brains. Such rerouting could provide a mechanistic hypothesis for extracellular tau release supporting the cell-to-cell propagation of tau.

### CMA deficiency accelerates tau spreading

**Fig. 6:**
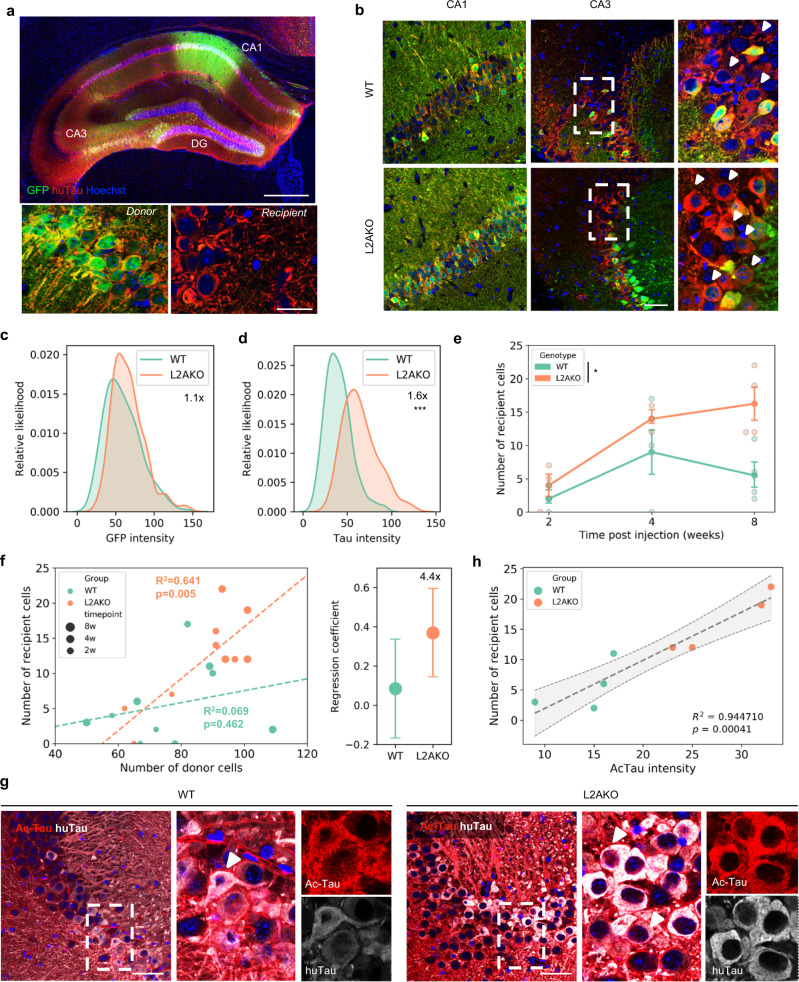
CMA deficiency accelerates Tau spreading. **a** Example of immunofluorescence for human tau (huTau-red) and GFP (Green) of the whole hippocampal formation in coronal section. Bottom pictures are representative pictures of donor [GFP+/huTau+] and recipient [GFP−/huTau+] cells. Scale bars: top: 100 µm; bottom 20 µm. *n* = 20 mice (10 WT and 10 L2AKO). **b** Representative images of Tau and GFP levels in CA1 (injection site) and CA3 (spreading site) in WT and L2AKO mice at 8 weeks after surgery. Scale bar: 50 µm. *n* = 4 mice per genotype. **c** Distribution of GFP intensity in donor cells (with kernel density estimation) in WT and L2AKO mice. **d** Distribution of human Tau intensity in donor cells (with kernel density estimation) in WT and L2AKO mice [Two-tailed *t*-test *t*_553_ = 16.113, *p* = 3.69 × 10^−48^]. **e** Number of recipient cells over time [Two-way ANOVA genotype effect: *F*_(1,14) _= 7.668, *p* = 0.0151]. *n* = 3, 3, and 4 mice per genotype at 2, 4, and 8 weeks, respectively. **f** Left: scatterplot showing the average number of recipient versus donor cells per mouse. Dotted lines are the linear regression per genotype. Right: slopes of the linear regression between recipient and donor cells per genotype. *n* = 3, 3, and 4 mice per genotype at 2, 4, and 8 weeks, respectively. **h** Scatterplot showing the average intensity of acetylated tau in donor cells versus the average number of recipient cells per mouse. The dotted line is the mean linear regression +/−95% confidence interval (gray zone). **g** Levels of human tau (huTau) and K174 acetylated tau (ac-Tau) in recipient cells between WT and L2AKO mice. *n* = 4 mice per genotype. Scale bar: 50 µm. All values are mean ± s.e.m. except otherwise specified. Differences with WT mice were significant for *p < 0.05, ***p < 0.0005.

To experimentally test whether CMA deficiency, as the one observed in AD patients, increases tau spreading in vivo, we took advantage of a recently developed adeno-associated virus (AAV) expressing green fluorescent protein (GFP)-2a-human tau (huTau)^58,59^ that generates equimolar amounts of GFP and human tau upon self-cleavage. Following unilateral injection in CA1, we could identify, by immunofluorescence, transduced neurons (donor cells [GFP^+^/huTau^+^]) and neurons that received huTau through cell-to-cell propagation (recipient cells [GFP^−^/huTau^+^] (Fig. 6a). Transduction was mostly neuronal as no huTau signal was observed colocalizing with astrocytes or microglia markers (Supplemental Fig. 6a). Recipient neurons were observed throughout the hippocampal formation suggesting a mixed synaptic and non-synaptic (global) transmission of tau^59^.

When we injected WT and CMA-deficient (L2AKO) mice and collected brain tissue after 2, 4, and 8 weeks following injection (Fig. 6b and Supplemental Fig. 6b, c), we observed a strong accumulation of huTau (1.6 folds) compared to GFP (1.1 folds) in L2AKO mice, thus confirming the contribution of CMA to tau clearance (Fig. 6c, d). Despite similar numbers of donor cells and tau expression (Supplemental Fig. 6b, c), the number of recipient cells was also significantly higher in L2AKO mice compared to WT mice, especially at the latest timepoint (Fig. 6e). In fact, the correlation between the numbers of recipient neurons and donor neurons in a given genotype showed a steeper slope (4.4 folds) in L2AKO mice compared to WT mice (Fig. 6f), supporting those donor neurons in L2AKO mice are more prone to spread. Direct analysis of acetylated tau in the injected brains, showed accumulation of acetylated tau in both donor and recipient neurons in CMA-deficient mice (Fig. 6g and Supplemental Fig. 6d–f). Furthermore, we observed a strong relationship between levels of acetylated tau in donor neurons and the number of neurons that become positive for tau through spreading (recipient neurons) (Fig. 6h), thus suggesting that elevation of intracellular acetylated tau predicts tau propagation in this mouse model of tauopathy.

Overall, our findings highlight a complex interplay of acetylated tau with different autophagic pathways, reveal a toxic effect of this modification on CMA function with the possible rerouting of acetylated tau towards other selective autophagy pathways in disease brains, ultimately leading to enhanced extracellular release and cell-to-cell propagation.

## Discussion

In this study, we have characterized the contribution of CMA to tau degradation in vivo and discovered how this process is severely blunted upon tau acetylation. Acetylated tau, under normal conditions, is efficiently cleared up by macroautophagy, but a fraction of acetylated tau targeted to CMA exerts an inhibitory effect on this selective type of autophagy. We show here that CMA blockage favors rerouting of oligomers of acetylated tau toward LE/MVB by e-MI and that this mechanism could lead to extracellular release and cell-to-cell propagation of tau. Although the tau propagation studies in this work were performed in a mouse model of tauopathy, the fact that the same molecular mechanisms behind the toxicity of acetylated tau on CMA and the accumulation of oligomeric acetylated tau in LE/MVB can be detected in brains from AD patients highlights CMA disruption as a previously unknown common feature of the AD brains that could also contribute to neuronal degeneration and disease progression.

We identified a pH dependence for internalization into lysosomes via CMA, unique for tau, that explains its highly efficient degradation through CMA under normal conditions. So far, we have not observed such a feature in any of the known CMA substrates, but future studies will determine if pH-dependent binding to hsc70 is a shared feature of a subset of CMA substrates. The mechanisms behind the toxic effect of acetylated tau on CMA are also different from the one described before for other neurodegeneration-related pathogenic proteins, such as alpha-synuclein^60^ and leucine-rich repeat kinase 2^61^. These proteins disrupt CMA by preventing the formation of the CMA translocation complex. In contrast, assembly of the multimeric L2A complex and translocation of pathogenic tau is initiated normally, but it is halted by loss of the pH-dependent interaction with hsc70. This result suggests that conditions that compromise lysosomal acidification, such as those described in familial AD^36,57^ could also have a negative impact on the degradation of tau by CMA. Therefore, restoring lysosomal acidification could help reinstate normal tau degradation by CMA, as long as the CMA blockage is reversible. Our in vitro studies with chemical hsc70 activators to enhance its disassembling effect on the L2A multimeric complex^41^ support CMA blockage reversibility. However, the efficiency of such types of approaches in the AD brain after chronically sustained disruption of CMA by acetylated tau could be different.

The combined analysis of unmodified tau degradation with that of the acetylation-mimetic variants supports that acetylation (K^174^ or K^274^) favors the degradation of tau through macroautophagy. Acetylated tau seems to be targeted to autophagosomes actively instead of through in bulk sequestration because levels of acetylated tau are almost undetectable in the cytosol but highly enriched in autophagosomes (Fig. 1f). Selective degradation of proteins by macroautophagy occurs often through aggrephagy, which first requires aggregation and subsequent recognition by specific macroautophagy receptors. Selective targeting to macroautophagy upon acetylation has also been described for huntingtin through promoting its organization into large complexes recognized by the autophagy receptor p62^62^. However, we did not detect the organization of acetylated tau in aggregates or oligomers inside autophagosomes, suggesting that it is not a prerequisite for its macroautophagy degradation. Future studies are needed to identify the molecular components that allow for selective macroautophagy of acetylated tau.

Although acetylated tau seems preferentially degraded through macroautophagy in steady-state conditions, its accumulation in L2AKO brains implies a role for CMA in regulating its intracellular levels. Since acetylation is dynamic and the fraction of acetylated tau may be dependent on total tau levels, acetylation could occur secondary to the CMA blockage and accumulation of unmodified tau. In this context, a reduction in CMA activity as the one described in aging may trigger the accumulation of the forms of acetylated tau that we demonstrate to be toxic for CMA, further perpetuating reduced CMA activity. It is also possible that some acetylated forms of tau are targeted to CMA-active lysosomes. For example, the fact that K^174^ tau was more abundant in CMA-active lysosomes and blockage of CMA increased its abundance in macroautophagy-engaged lysosomes, supporting possible rerouting of acetylated variants from one autophagic pathway to another. Although we did not find that K^174^ acetylation generated a CMA-targeting motif in tau by mimicking the biochemical properties of glutamine, as described for other substrates^63^, it is possible that acetylation of this residue alters tau structure thus exposing its canonical CMA-targeting motifs^29^.

Interestingly, in contrast to the reduced degradation of acetylated tau by CMA, we found that this form of the protein was more readily degraded by e-MI, even when in oligomeric form since e-MI does not need substrate unfolding as a prerequisite^43^. Rerouting to e-MI might allow acetylated tau to bypass the steps that presented a challenge for its degradation through CMA, making removal of an oligomeric complex of acetylated tau from the cytosol more favorable. How hsc70 triages proteins for CMA or e-MI is still poorly understood. However, K^274^ acetylation, recently shown to increase tau interaction with hsc70^64^, showed no preference for CMA-active lysosomes but a high enrichment in LE/MVB, suggesting that post-translational modifications in the substrates and/or their ability to unfold may determine binding affinity for hsc70 and the compartment where the chaperone targets them. Since the amount of acetylated tau in diseased brain is dynamic and would differ greatly in neurons at different disease stages, future studies directly determining the percentage of total tau acetylated at each stage should provide key information to estimate the contribution of the observed re-routing of tau to LE/MVB in disease progression. In light of our findings showing that levels of specific acetylation on tau (i.e., ac-K174) also differ greatly in different subcellular compartments, quantitative analysis of the percentage of acetylated tau will need to be performed at the subcellular level.

Cell-to-cell propagation of tau is thought to underlie pathology spreading in the brain, however, the mechanism(s) for tau secretion is poorly understood^65^. We demonstrate that e-MI is efficient in the degradation of acetylated tau and that it also allows its extracellular release. This dual function stems from the fact that LE/MVB can degrade proteins directly or upon fusion with lysosomes, but they can also fuse with the plasma membrane and release their luminal content (exosomes)^65^. The small amount of extracellular tau upon e-MI or MA blockage suggests that these pathways contribute to tau release in basal conditions^23^. Exacerbated release through LE/MVB is also the mechanism behind the striking increase in extracellular tau observed upon CMA blockage. Our data using the GFP-2a-huTau construct confirms that this increased secretion in CMA-deficient animals is accompanied by uptake in neighboring neurons and acceleration of spreading in the brain. This observation makes attractive the idea that the previously described decline of CMA activity with age^66^ could be behind the recently described accelerated spreading of tau in aging^59^. Future studies are now required to assess if tau propagation in the aging brain involves rerouting of tau towards e-MI and if the spreading observed here for P301L tau, a mutation associated with FTD but not AD^67^ is also reproduced for other forms of tau.

The isolation of autophagy/lysosomal compartments from AD patient brains performed in this work provides, for the first time, information on the forms of tau associated with each of the compartments and the changes in this association with disease. Functional studies with these human brain fractions allowed us to directly demonstrate loss of CMA activity in AD brain lysosomes that likely occur through a similar mechanism (disruption of LAMP2A dynamics) to the one observed in our experimental mouse models. One caveat of using human samples with advanced AD pathology is that at late stages of the disease some vulnerable neurons are already dead and therefore the signal that is captured can arise from less vulnerable neurons and glia. To minimize this problem, we selected the middle frontal gyrus because this brain region displayed a low range of atrophy (around 10%) compared to other brain regions such as the hippocampus or the entorhinal cortex (20 to 30%)^68,69^. However, future studies at different stages of the disease and in other brain regions are needed to better delineate the evolution of temporo-spatial changes in autophagy in AD.

In summary, our study reveals a complex interplay of acetylated tau with different autophagic pathways that contributes to fine-tuning its intracellular levels. The inhibitory effect of acetylated tau on CMA suggests that blockage of this selective form of autophagy may contribute to neuronal toxicity and disease progression. Therefore, enhancing CMA activity or preventing the arrival of toxic forms of tau to CMA-active lysosomes, or promoting degradation of rerouted tau inside LE/MVB could all be fruitful options to prevent neurodegeneration in AD and other tauopathies.

## Methods

### Animals and cells

Adult male C57BL/6 mice were from Jackson Laboratory and used under an animal study protocol approved by the Institutional Animal Care and Use Committee (IACUC) of the Albert Einstein College of Medicine. The L2A and Atg7 knockout mouse models were generated by crossing L2A^flox/flox^ mice^70^ or Atg7^flox/flox^ mice^71^ with CamKinase II-Cre mice. Wild-type littermates were used as control. Animals were maintained at 19–23 °C 30–60% relative humidity and timer-controlled 12 h light/dark cycle. CSF was isolated from the cisterna magna of controls and L2AKO mice using a glass capillary with negative pressure connected to a micromanipulator to minimize blood contamination^72^. The mouse neuroblastoma cell line Neuro-2a (N2a) was a gift from Drs. Mandelkow (DZNE, Germany), mouse embryonic fibroblasts (MEFs) from wild-type (WT) or *Atg5*^−/−^ (KO) mice were a gift from Dr. N. Mizushima (The University of Tokyo). MEFs from wild-type (WT) mice were generated in our labortatory^73^. Cells knocked down for VPS4, were generated using the small interference RNA (siRNA) from the Mission-Sigma library (Sigma-Aldrich) VPS4A (TRCN0000101417), VPS4B (TRCN0000101821)^43^. Cells knocked down for Atg7 and LAMP2A were generated using shRNA using the following sequences: ATG7: 5′-GATCCCCGCAGCTCATTGATAACCATTTCAAGAGAATGGTTATCAATGAGCTGCTTTTTC-3′ (for Atg7) and 5′-CACCGCTGCAATCTGATTGATTATCGAAATAATCAATCAGATTGCAG-3′ (for LAMP2A)^43,74^. Cells were transfected using Lipofectamine RNAiMAX Transfection Reagent (Invitrogen) in Opti-MEM media without antibiotics. Cells were maintained in Dulbecco’s modified Eagle’s medium (DMEM) (Sigma), in the presence of 10% fetal bovine serum (FBS), 50 μg/ml penicillin, and 50 μg/ml streptomycin at 37 °C with 5% CO_2_.

Transcription factor-mediated human neuron differentiation was induced from a wildtype iPSC line with stable neurogenin 2 integration^21^. We followed a two-step protocol in which cells were transfected using Lipofectamine Stem (Invitrogen) and transgenic cell populations were then selected and enriched through cell-surface affinity to magnetic streptavidin beads of a streptavidin binding peptide fused to the low-affinity nerve growth factor receptor^75^ and generated 8 weeks old wildtype human neurons to detect tau release.

### Human brain samples

Middle frontal gyri from European American brains were obtained from the Charles F. and Joanne Knight Alzheimer’s Disease Research Center. AD pathology was measured using Braak and Braak staging. The Washington University IRB reviewed the Knight ADRC Neuropathology Core (from where the brains were obtained) operating protocol, as well as this specific study, and determined it was exempt from approval. In the state of Missouri, individuals can give prospective consent for autopsy. Our participants provide this consent by signing the hospital’s autopsy form. If the participant does not provide future consent before death the DPOA or next of kin provide it after death. The age of donors ranged from 70-80 years with an average age of 77.8 ± 3.2 years in controls and from 73 to 95 years with an average age of 82.1 ± 2.1 years in AD patients. The percentage of females was 43% in control and 60% in AD patients, and the average postmortem interval of 12.5 ± 2.0 and 10.1 ± 1.2 h in control and AD patients, respectively. The amount of tissue from each donor determined whether the sample was used for isolation of autophagic vacuoles, lysosomes, or late endosomes (although in most cases the three samples could be prepared). Those samples with not enough tissue for isolation contributed to some of the biochemical analysis with brain homogenates. Recruitment was not done as part of this study and selection of the brain tissue from the repository was done on the basis of the histopathology report (that assigned brain tissue as healthy control or AD pathology) and the postmortem time (within the reported range) in order to keep samples comparable. We are not aware of any bias in the selection of tissues that could impact the results presented.

### Chemicals and plasmids

Sources of reagents and chemicals were as described before^50,74,76^. The antibodies against mouse LAMP-1 (clone 1D4B; 1:5000), human LAMP-1 (clone H4A3; 1:5000), human LAMP-2 (clone H4B4; 1:5000), were from the Developmental Hybridoma Bank (University of Iowa), against human LAMP-2A (#ab18528; 1:5000) and GAPDH (#ab8245; 1:5000) from Abcam, against GST (#136700; 1:1000) from Invitrogen, against hsp90 (#adi-spa-835-f; 1:1000) from Stressgen, against actin (#A2066; 1:5000), FLAG (#F4042; 1:1000) and Vps4 (#sab4200025; 1:1000) from Sigma, against light chain 3 protein (LC3B, #2775, 1:1000), p53 (#2524s; 1:1000), Rab9 (#5133; 1:1000) and Atg7 (#2631; 1:1000) from Cell Signaling, against GFP (#ta150052; 1:1000) from Origene, against hsc70 (clone 13D3, #NB120-2788; 1:1000) and Atg5 (#NB110-53818, 1:1000) from Novus, against p62 (#BML-PW9860-0100; 1:2000) from ENZO, against BiP (#610978; 1:1000) from BD Transduction, against EEA-1 (#610456; 1:1000) from BD Biosciences, against NBR-1 (#H00004077-B01P; 1:1000) from Abnova. The antibody against mouse LAMP-2A (1:7000) was developed in our laboratory^40^. The antibodies against total tau (DA9, 1:5000) and phosphorylated tau (PHF-1, 1:1000) were a gift from Dr. P. Davies (The Feinstein Institute). The antibodies against acetylated tau in lysines 174 (K174, 1:500) and 274 (K274, 1:500) were developed in Dr. Li Gan’s laboratory^13,77^. The antibody against human oligomeric tau (T22, #ABN454, 1:1000) was from EMD Millipore. The antibody against Mucolipin was a gift from Dr. R. Nixon (NYU). Human tau cDNA (1N4R) in the pRK172 vector for purification of recombinant tau was a gift from Dr. Mucke lab, Gladstone Institutes. The mCherry-GFP-LC3 was generated by inserting the coding sequence^44^ (1,1Kb) in the AtR2 and AtR1 sites of the pLX-301 plasmid using the TOPO PCR Cloning kit (Invitrogen) with the following oligonucleotide primers: 5’ CACTGACAATTTCATCCCGAACGTCTCCTGGGAGG 3’ (for Topo-R) and 5’ CACCATGGTGAGCAAGGGCGAGGAGGAC 3’ (for Topo-D). Plasmids for WT tau, tau K274,281Q^12^ were generated by site-directed mutagenesis (QuickChange kit from Agilent) in Human WT tau cDNA cloned into the pcDNA3.1 vector (Invitrogen) and site-directed mutagenesis (QuickChange kit from Agilent) was used to generate tau K274,281Q by introducing A820C and A841C mutations with the following oligonucleotide sequence: 5’ GGAGGCGGGCA GGTGCAGATAATTAATAAGCAGCTGGAT 3’. The eGFP-2a-huTau construct was cloned in a pseudotype 2/8 AAV backbone, for efficient neuronal tropism in the central nervous system, under the chicken beta-actin (CBA) promotor^58^. The vector also contains a woodchuck hepatitis virus posttranscriptional regulatory element (WPRE – for long-term stable expression) and polyA sequence downstream of the target gene. The AAV was produced at a titer of ~0.6×10^13^ infectious particles/mL at the Mass Eye and Ear (MEEI) vector core (Boston, MA, USA).

### Tau purification and acetylation

Human tau cDNA (1N4R) was expressed in BL21 (DE3) E. Coli and purified by ion-exchange chromatography. Briefly, pelleted bacteria were resuspended in PIPES buffer (50 mM PIPES, 1 mM EGTA, a protease inhibitor (Sigma), pH 6.8), sonicated, and centrifuged at 27,000 × *g* for 15 min. The supernatant was boiled at 95 °C for 10 min and after centrifugation at 100,000 × *g* for 15 min, it was loaded into a column with P11 phosphocellulose resin (Whatman) and washed with 0.1 M NaCl in PIPES buffer, followed by elution with 0.3 M NaCl in PIPES buffer. The purified recombinant tau proteins were assessed by coomassie blue-stained SDS-polyacrylamide gel electrophoresis. In vitro acetylation reaction was performed as previously described^13^ with minor modifications. Briefly, 1 μg of human recombinant tau, 2 μM of acetyl-CoA (Sigma), and 100 ng of purified GST-p300 in acetylation buffer (50 mM HEPES, pH 8.0, 10% glycerol, 1 mM dithiothreitol (DTT), and 10 mM sodium butyrate) were incubated 4 h at 30 °C with constant shaking. Control non-acetylated tau in these studies was incubated in the same buffer but in absence of the GST-p300 enzyme.

### Protein purification

Hsc70 and DJA2 were expressed and purified using previously reported methods^78^. Nucleotide free Hsc70 was prepared from several days of dialysis in assay buffer (0.017% Triton X-100, 100 mM Tris-HCl, 20 mM KCl, and 6 mM MgCl_2_, pH 7.4) at 4 °C to remove nucleotide. For tau purifications, the previously reported protocol^79^ was used with the following modifications. Sodium chloride (500 mM) and a chemical chaperone, betaine (10 mM), were included in the growth media prior to induction to improve expression and minimize degradation products. Expression was induced with 200 µM IPTG for 3.5 h at 30 °C and purified using cation chromatography. Protein purity was confirmed by SDS-PAGE analysis.

### Tau binding ELISA

The method was adapted from a previous report^80^. Briefly, 1 µM human hsc70 (30 µL) was immobilized overnight at 37 °C in clear, non-sterile 96-well plates (Thermo) in 50 mM MES (pH 5.5) and 0.5 mM DTT with 1 mM ADP. Wells were washed with 100 µL of PSB-T (3 × 3 min., rocking) prior to the addition of 30 µL of 4R0N tau or K280Q tau solution in binding buffer (40 mM KCl, 8 mM MgCl_2_, 100 mM NaCl, 0.5 mM DTT, 0.01% Tween, and 25 mM acetate for pH 5, 25 mM MES for pH 6, 25 mM HEPES for pH 7.4, and 25 mM Tris for pH 8) with 1 mM ADP for 3 h at RT. After blocking in 5% milk, quantification of tau binding was performed using rabbit anti-tau (H150) primary antibody (Santa Cruz, sc-5587, 1:2000 in TBS-T, 50 µL/well) and goat anti-rabbit HRP-conjugated secondary (Anaspec, 28177, 1:2000 in TBS-T, 50 µL/well). TMB substrate (Cell Signaling, 7400 L) and 1 N HCl were used to detect binding. Absorbance was measured using a SpectraMax plate reader (OD_450_). Minimal, non-specific binding of tau to empty wells was subtracted as background, and curves were fit using non-zero intercept hyperbolic fits in Prism (GraphPad Software).

### ATPase assay

The assay was adapted from previously reported methods^81^. Briefly, hsc70 (1 μM), 1 mM ATP, and increasing amounts of DJA2 were incubated for 1–2 h in assay buffer (0.017% Triton X-100, 20 mM KCl, and 6 mM MgCl_2_, with 100 mM acetate for pH 5, 100 mM HEPES for pH 7, and 100 mM Tris-HCl for pH 7.4 and pH 8). Afterward, 80 µL of malachite green reagent was added for phosphate detection followed by the addition of sodium citrate to halt non-enzymatic ATP hydrolysis. Absorbance was measured by a SpectraMax plate reader (OD_620_) to determine phosphate concentration.

### Isolation of subcellular fractions

*CMA active lysosomes*. Lysosomes with high activity for CMA were isolated from rat liver, mouse brain, and from control and AD patients brains by centrifugation of a light mitochondrial-lysosomal fraction in a discontinuous metrizamide density gradient^45^. Homogenates were subjected to low-speed centrifugation at 6800 × *g* for 5 min using a fixed angle rotor and the supernatant was further centrifuged at 17,000 g for 10 min to obtain a pellet enriched in light mitochondria and lysosomes. This pellet was washed in an excess of 0.25 M sucrose and subjected to a second 17,000 × *g* spin for 10 min. The resulting pellet was resuspended in 1.1 ml of 0.25 M sucrose and 2.2 ml of 85.6% stock metrizamide solution (final concentration: 57% metrizamide). This pellet mixture was loaded at the bottom of an ultracentrifuge tube (14 × 89 mm, Beckman ultra-clear centrifuge tube) and overlaid with a discontinuous metrizamide step-gradient in the following order: 2 ml of 32.8% metrizamide, 3.3 ml of 26.3% metrizamide, and 3.6 ml of 19.8% metrizamide and was filled to the top with 0.25 M sucrose. Centrifugation was carried out in a Beckman Coulter Optima XL-100K centrifuge using an SW41 rotor (Beckman) at 141,000 × *g* for 1 h (acceleration: 170 rpm/3 mins, deceleration: 170 rpm/4 mins). After centrifugation, four separate bands (P1-4) were enriched in the following (from top to bottom): P1-lysosomes active for CMA (CMA + lysosomes); P2-a mix of lysosomes active and non-active for CMA (CMA + and CMA− lysosomes); P3-light mitochondria and lysosomes; P4-light mitochondria. CMA+ and CMA− lysosomes were obtained by collecting P1 and P2 separately and pelleting at 37,000 × *g* for 15 min in 10 volumes of 0.25 M sucrose in a Beckman Allegra 64 R centrifuge, Then, the P2 pellet was resuspended in MOPS/Sucrose buffer and centrifuged at 10,000 × *g* for 5 min at 4 °C in an Eppendorf 5415C tabletop centrifuge. This procedure separated CMA+ lysosomes (retained in the supernatant) and CMA− lysosomes (pelleted down). This supernatant was used to resuspend the P1 pellet. The CMA− lysosomes were resuspended in an equivalent volume to the CMA+ lysosomes.

Cytosolic fractions were obtained by centrifugation for 1 h at 100,000 × *g* of the supernatant obtained after separating the mitochondria-lysosome-enriched fraction (the first 17,000 × *g* spin).

*Autophagic vacuoles.* Fractions enriched in autophagic vacuoles were isolated from mouse brains by centrifugation in a discontinuous metrizamide density gradient by a modified method described before^82^. Brain homogenates were initially centrifuged at 2000 × *g* for 5 mins in a Beckman Allegra 64 R centrifuge (F0630 rotor) to pellet nuclei, unbroken cells, and plasma membrane, which was resuspended in 0.25 M sucrose and centrifuged again in the same way. The ensuing supernatants for each of these spins were then combined and subjected to centrifugation at 17,000 × *g* for 12 mins. The resulting pellet was washed with sucrose and the same spin was repeated. After this wash, the pellet was resuspended in 0.95 ml of 0.25 M sucrose and adjusted to a final concentration of 51% metrizamide upon addition of 1.4 ml of 85.6% stock. This mixture was loaded at the bottom of an ultra-clear centrifuge tube (14 × 89 mm, Beckman) and layered with the following discontinuous metrizamide gradients: 4 ml of 26% metrizamide, 2 ml of 24%, 2 ml of 20%, and 2 ml of 15%. The gradient was topped with 0.25 M sucrose and centrifuged in the Beckman Coulter Optima XL-100 K Ultracentrifuge for 3 h 9 min at 105,000 × *g* (SW41 rotor, acceleration: 170 rpm/4 min, deceleration: 170 rpm/4 min). After this spin, four bands were visible, at the interphases enriched in the following fractions (starting at the top): fraction 1-AV10 (APG), fraction 2-AV20 (autolysosomes), fraction 3-LysC (low CMA activity), fraction 4-light mitochondria. Finally, these bands were collected and centrifuged at 24,000 × *g* for 10 mins in 10 volumes of 0.25 M sucrose in the Beckman Allegra 64 R centrifuge (F0630 rotor) and the resulting pellets were resuspended in 0.25 M sucrose.

*Late endosomes.* Isolation of brain late endosomes was obtained by centrifugation of a mitochondrial-lysosomes-endosomal fraction in two consecutive discontinuous Sucrose/Percoll gradients laid over a 2.5 M cushion by a modified method described before^83^. Homogenates from animal brains obtained in 0.25 M Sucrose–1 mM EDTA, processed as described above, were subjected to low-speed centrifugation at 2000 × *g* for 5 min in an F0630 rotor in a Beckman Allegra 64R centrifuge to sediment nuclei and unbroken cells, plasma membrane, and heavy mitochondria. The supernatant was decanted and overlaid on top of the ultracentrifuge tube (14 × 89 mm Beckman ultra-clear centrifuge tube) on a discontinuous sucrose gradient as follows: 1 ml of 2.5 M sucrose, 9 ml of 27% Percoll in SE buffer. Centrifugation was carried out in an SW41 rotor (Beckman) at 33,000 × *g* for 1 h and 5 min at 4 °C (acceleration: 170 rpm/3 min and deceleration: 170 rpm/4 min) using a Beckman Coulter Optima XL-100K ultracentrifuge. Approximately 1 ml of the brown band (Golgi, late endosomes, and early endosomes mixture) from the top of the 27% Percoll fraction was collected and diluted with 1 ml of SE and overlaid on top of a discontinuous sucrose step-gradient as follows: 1 ml 2.5 M sucrose, 9 ml 10% Percoll in SE buffer. Centrifugation was carried out in an SW41 rotor (Beckman) at 33,000 × *g* for 1 h and 5 min at 4 °C (acceleration: 170 rpm/3 min and deceleration: 170 rpm/4 min) using a Beckman Coulter Optima XL-100K ultracentrifuge. The top of 10% Percoll (from the second gradient) was collected as Golgi and early endosomes and the interface between 27% Percoll SE and 2.5 M sucrose (from the first gradient) was collected as late endosomes. Both fractions were pelleted twice at 100,000 × *g* for 10 min at 4 °C. The pellets were frozen at −80 °C or resuspended in appropriate volume of 0.25 M sucrose for further experimentation.

*Lysosomal membranes*. Isolation of lysosomal membranes was done by disruption of lysosomes through a hypotonic shock followed by 10 freeze-thaw cycles in the presence of protease inhibitors. Matrix was recovered in the supernatant upon centrifugation at 250,000 g for 1 h.

### Intracellular protein turnover

To measure the degradation of long-lived proteins, confluent cells were labeled with ^3^H-leucine (2 μCi/ml) for 48 h at 37 °C, transfected with the indicated plasmids and then extensively washed and maintained in complete (10% FBS) or serum-deprived media containing an excess of unlabeled leucine (2.8 mM) to prevent reutilization of radiolabeled leucine^47^. Aliquots of the media taken at different times were precipitated with TCA and proteolysis was measured as the percentage of the initial acid-insoluble radioactivity (protein) transformed into acid-soluble radioactivity (amino acids and small peptides) at the end of the incubation. Total radioactivity incorporated into cellular proteins was determined as the amount of acid-precipitable radioactivity in labeled cells immediately after washing.

### Measurement of lysosomal activity

CMA activity in vitro was measured using isolated intact lysosomes incubated with purified proteins and subjected to immunoblot^47^. Binding was calculated as the amount of substrate protein bound to the lysosomal membrane in the absence of protease inhibitors and uptake by subtracting the amount of protein associated with lysosomes in the presence (protein bound to the lysosomal membrane and taken up by lysosomes) and absence (protein bound to the lysosomal membrane) of protease inhibitors.

CMA activity in intact cells was measured using lentivirus-mediated expression of the KFERQ-PS-Dendra2 and high-content microscopy^49^. Cells were plated in a 96-well plate and photoactivated with a 405 nm light-emitting diode (LED: Norlux) for 4 min with the intensity of 3.5 mA (current constant). After 16 h, cells were fixed with 4% paraformaldehyde, and images were captured with a high-content microscope (Operetta system, Perkin Elmer) and quantification was performed with the manufacturer’s software in a minimum of 800 cells (approx. 9 fields).

*LAMP-2A dynamics*. Multimerization of LAMP-2A at the lysosomal membrane was studied on 3–12% NativePAGE Bis-Tris Gels (Invitrogen) after solubilization in 1% octylglucoside (in 20 mM MOPS and 150 mM NaCl buffer)^41^.

### Measurement of endosomal microautophagy activity

e-MI activity in vitro was measured using isolated late endosomes incubated with purified proteins and subjected to immunoblot^43^. Binding and internalization were calculated as the amount of substrate protein bound to the late endosomal membrane and intact internal vesicles in the absence of protease inhibitors and luminal degradation by subtracting the amount of protein associated with late endosomes in the presence (protein bound to the endosomal membrane, intact internal vesicles and inside late endosomal lumen) and absence (protein bound to the endosomal membrane and intact internal vesicles) of protease inhibitors.

### Intracranial injections

Adeno-associated viruses pseudotype 2/8 (AAV2/8) encoding eGFP-2a-huTau^P301L^ under the chicken β actin (CBA) were cloned and produced at a titer ~0.6 × 10^13^ virus particles/mL^59^. AAVs were injected unilaterally in the right hippocampus above the CA1 region (A/P −2.0 mm, M/L: ± 1.5 mm, D/V from brain surface −1.5 mm) under standard aseptic surgery. One microliter of the virus was injected using 34-gauge 10 µl Nanofil (WPI, USA) syringe and slowly infused at 0.2 µl/min using a UMP-III micropump (WPI, USA). At completion, the needle was left in place for 5 min to allow diffusion of the viral solution and avoid leakage.

### Immunofluorescence

For immunostaining of acetylated tau (anti-acK174), in order to reduce the nonspecific nuclear staining, we first pre-absorbed the antibody by acetone Tau-knockout (KO) mice brain powder^13^. Briefly, Tau KO mouse brains were first rinsed in 0.8% NaCl in Ca^2+^/Mg^2+^ free PBS, homogenized in 100 mM MES, 1 mM EGTA, 0.5 mM MgCl_2_, 0.25 mM GTP at pH6.5, filtered through the ceramic filter, acetone-washed, and dried overnight. Mouse brains were sectioned into 10-µm slices on a Leica CM1900 Cryostat. Antigen retrieval was first performed by heating the sections in 10 mM citric acid at a high-pressure cooker for 5 min. The brain slices were then permeabilized in Tris-buffered saline (TBS) with 0.5% Triton X-100 and blocked with 10% normal goat serum. For 3,3′-diaminobenzidine (DAB) immunohistochemical staining, sections were quenched to suppress endogenous peroxidase activity, followed by incubation with AC312. Immunoreactivity was detected with 0.25 mg/ml DAB with 0.01% H_2_O_2_. Images were acquired on a bright-field Leica DM5000B microscope with a Leica DFC310 FX camera.

For quantification of tau spreading, mice were perfused with 0.9% sodium chloride at 2, 4, and 8 weeks after surgery. The whole brain was extracted and post-fixed overnight in 4% paraformaldehyde, and then cryoprotected in 20% sucrose/PBS. Brains were flash-frozen in isopentane, cut coronally at 40 µm in Leica CM cryostat, and stored in PBS-0.2% sodium azide at 4 °C until processing. For immunostaining, the floating brain sections were washed briefly in PBS and then permeabilized with 0.2% Triton X-100/TBS for 20 min at room temperature, blocked in 5% normal goat serum (NGS)/PBS for 1 h at room temperature, and then incubated with primary antibodies diluted in 3% NGS/PBS overnight at 4 °C: mouse anti-human tau Tau13 (1:1000, abcam #ab19030), rabbit anti-human tau TauY9 (1:1000, Enzo Life Science), rabbit anti-mouse GFAP (1:1000, abcam #ab5804), goat anti-mouse Iba1 (1:1000, abcam #ab5076). After washing three times with PBS, secondary antibodies were diluted in 3% NGS/PBS and applied for 1 h at room temperature: Alexa-555 anti-mouse, Alexa-555 anti-rabbit, Alexa 647 anti-mouse, and Alexa 647 anti-goat (1:2000, Thermo Fisher Scientific). After three washes in PBS, sections were stained with Hoechst33342 (1:2000, Thermo Fisher Scientific) for 2 min and then mounted on microscope glass slides with ProLong Diamond mounting media (Thermo Fisher Scientific). Images were acquired with a Leica confocal TCS-SP8 (Leica Microsystem) using the Leica LAS-X software for image adquisition and ImageJ Software (v.2.1.0) (NIH) was used for image processing and analysis. Numbers of donor and recipient cells were manually counted in the ipsilateral hippocampal formation by an experimenter blind to genotypes.

### RNA isolation and qPCR analyses

Total RNA was isolated using the RNeasy Plus kit (Qiagen) or the RecoverAll Total Nucleic Acid Isolation Kit for FFPE (Thermo Fisher Scientific) according to the manufacturer’s instructions. For RNA extracted from tissue sections, three 40µm-thick sections were selected per animal and the right (injected) hippocampus was manually dissected. Total RNA was reverse transcribed into cDNA using Superscript II (Invitrogen), and quantitative RT-PCR analyses were performed using Podwer SYBR Green PCR Mix (Applied Biosystems) on a StepOne Plus Real-Time PCR System (Applied Biosystems) using the primers shown in Supplementary Table 1. Thermal cycling conditions used were according to the instruction of the SYBR Green mix protocol, and relative RNA abundance was calculated using the comparative 2^−∆∆Ct^ method^84^. Water was used as the negative control for the qPCR analysis. All reactions were performed in triplicates.

### Electron microscopy

Isolated organelles were fixed in 2.5% glutaraldehyde in 100 mM sodium cacodylate, pH 7.43 maintained isomolar by addition of 0.25 M sucrose. Samples were post-fixed in 1% osmium tetroxide in 100 mM sodium cacodylate, pH 7.43, followed by 1% uranyl acetate. After ethanol dehydration and embedment in LX112 resin (LADD Research Industries, 21210), ultrathin sections were cut on a Reichert Ultracut E and were stained with uranyl acetate followed by lead citrate. All grids were viewed on a JEOL 100CX II transmission electron microscope at 80 kV.

### General methods

Protein concentration was determined using the Lowry method with bovine serum album as a standard^85^. Cells were solubilized on ice with RIPA buffer (1% Triton X-100, 1% sodium deoxycholate, 0.1% SDS, 0.15 M NaCl, 0.01 M sodium phosphate, pH 7.2). Tissues were resuspended in 0.25 M sucrose buffer, containing protease, phosphatase, and deacetylase inhibitors. Immunoblotting was performed after transferring SDS-PAGE gels to nitrocellulose membranes^86^. Efficacy and uniformity in the transference of proteins from the gel were confirmed using Ponceau red staining of the membranes. The proteins of interest were visualized by chemiluminescence using peroxidase-conjugated secondary antibodies in LAS-3000 Imaging System (Fujifilm). Densitometric quantification of the immunoblotted membranes was performed using Image J (NIH). When all samples from a single experiment could not be fit in a single gel, a fixed amount of the same sample was loaded at the beginning and end of each of the gels of the same set, and the average value of both flanking lines for each antibody was used for normalization when samples in different membranes were compared. A different grouping of samples/conditions was also used in the replicates for those experiments to avoid loading biases. Uncropped blots in the main and supplementary figures are shown in Supplemental Figs. 7 and 8, respectively.

### Statistical analysis, randomization, and sample size determination

All numerical results are reported as mean ± s.e.m. and represent data from a minimum of three independent experiments unless otherwise stated. No data exclusion was done in this study. In all instances n refers to individual experiments. Experiments in cells in culture or those involving isolation of intracellular organelles were performed on different days to confirm the reproducibility of the procedures. In addition, recovery and enrichment for each subcellular fractionation experiment were calculated to compare the purity and efficiency of isolations done on different days. All independent replications were successful. For the studies in live animals, the number of animals per group was determined through power analysis using the information from similar studies from our group or collaborators with similar methods^32,58–61,70^. For the studies performed in brain slices, cell lines in culture, and in vitro assays, we determine the number of experimental repetitions to account for technical variability and changes in culture conditions based on our previous studies using those systems^32,45,59–61^. For the studies using isolated organelles from animals, the number of specimens used was determined based on the average values of enrichment and recovery for the specific fraction using endogenous markers for each compartment from our previous studies.^45,60,61,70^. For the studies involving live animals, animals were randomly attributed to each surgery group according to their genotype using the SELECT BETWEEN RANGE function in Microsoft Excel. For the studies involving cells in culture or in vitro assays with purified organelles, treatment groups were attributed randomly between wells and plates to account for well or tube positioning effects. Immunofluorescence/immunohistochemistry of protein levels was performed blinded to their genotype or to the experimental group. For the morphometric analyses of the electron microscopy samples, annotated micrographs were performed blinded. For in vivo experiments, samples were blinded to the experimenter for the whole experiment, image acquisition, and data analysis. Blinding was lifted only for the final statistical analysis.

Basic data handling was performed using Microsoft Excel 365 (v.2101). Prior to statistical testing, normality was assessed using the Shapiro Wilk test. Parameters with two groups were compared using an unpaired, two-tailed t-test. Parameters with more than two groups were compared using one-way ANOVA and Tukey’s post-hoc analysis, Kruskal-Wallis test followed by Dunn’s post-hoc analysis or two-way ANOVA followed by Sidak post-hoc analysis with Prism software (v9-Graph Pad Software Inc) or using Python (Python software foundation v.3.7.4 available at https://www.python.org/) and the scientific python stack: scipy (v.1.3.1), numpy (v.1.17.2), and matplotlib (v.3.1.1). Statistical analysis was performed in all the assays, and significant differences are noted in the graphical representations.

### Reporting summary

Further information on research design is available in the Nature Research Reporting Summary linked to this article.

## Supplementary information

Supplementary Information

Reporting Summary

## Data Availability

There are no restrictions on data availability in this manuscript. Source data are provided with this paper.

## Source data

Source Data
