# Supplementary material for: Acetylated tau inhibits chaperone-mediated autophagy and promotes tau pathology propagation in mice
Source: Nat Commun. 2021 Apr 14;12:2238. doi: 10.1038/s41467-021-22501-9 (PMC8047017; doi:10.1038/s41467-021-22501-9)
Supplement: Supplementary file 1 — Supplementary Information [file 41467_2021_22501_MOESM1_ESM.pdf]

Supplementary Information

Acetylated tau inhibits chaperone-mediated autophagy and promotes tau pathology propagation in mice

Benjamin Caballero[1,2§#], Mathieu Bourdenx[1,2#], Enrique Luengo[1,2,3,4], Antonio Diaz[1,2], Peter Dongmin Sohn[5], Xu Chen[5], Chao Wang[5], Yves R. Juste[1,2], Susanne Wegmann[6,7], Bindi Patel[1,2], Zapporah T Young[8], Szu Yu Kuo[8], Jose Antonio Rodriguez-Navarro[1,2], Hao Shao[8,], Manuela G Lopez[3,4], Celeste M. Karch[9], Alison M. Goate[10], Jason E. Gestwicki[8], Bradley T. Hyman[6], Li Gan[5], and Ana Maria Cuervo[1,2*]

Supplementary Figures 1 – 8

Supplementary Table 1

Source Data

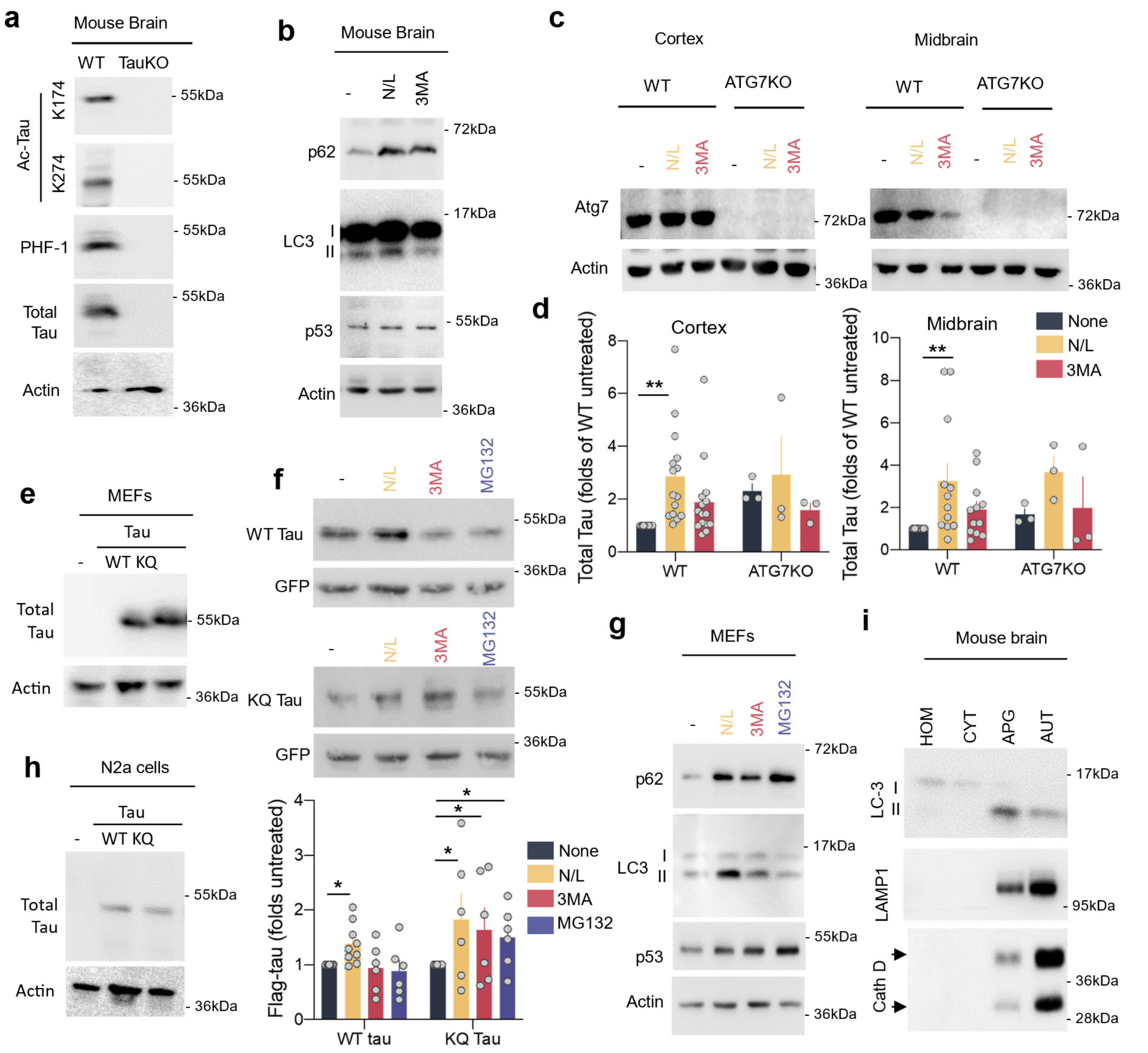

Supplemental Figure 1. Lysosomal degradation of acetylated tau. (**a**) Immunoblot for the indicated antibodies of homogenates of brains from wild-type (WT) and tau-null (TauKO) mice. Ac: acetylated. n=3 mice per genotype. (**b**) Immunoblot for the indicated antibodies of mouse brain slices incubated with the indicated inhibitors for 4h. n=3 mice. (**c**) Immunoblot for Atg7 of the indicated brain regions from wild-type (WT) and Atg7 knock-out (ATG7KO). n= 3 mice per genotype. (**d**) Changes of tau levels in brain slices from WT and ATG7KO cortex (left) [Two-way ANOVA Degradation pathway effect: $F_{(2,30)}$=5.014, p=0.0132] or midbrain (right) [Two-way ANOVA Degradation pathway effect: $F_{(2,42)}$=2.7, p=0.0785] incubated as in (b), n = 9 (cortex) and 13 (midbrain) slices for WT and 3 slices for ATG7 from 3 different mice. Representative immunoblots are shown in Fig. 1b. (**e**) Immunoblot for the indicated antibodies of mouse embryonic fibroblasts (MEFs) and Neuro-2a (N2a) cells transfected with unmodified FLAG-wild-type Tau (WT) or acetylation-mimetic FLAG-$K^{274,281}$Q tau (KQ). n=3 independent experiments. (**f**) MEF cells transfected with WT or KO tau and then treated with NH_4Cl and leupeptin (N/L), 3-methyladenine (3MA) or MG132 1μM for 4h or left untreated (-). Bottom: Quantification of tau levels under the different treatments. [Two-way ANOVA Degradation pathway effect: $F_{(2,24)}$=6.552, p=0.0054], n=9 (or None and N/L) and 6 (for 3-MA and MG-132) independent experiments (cell plating, treatments and cell collection performed on different days for each experiment). For samples run in different blots, a common lane of the same sample on each side was run in all of them and used for normalization purposes. Controls of inhibitors' efficiency are shown in (**g**). (**h**) Immunoblot for the indicated antibodies in Neuro-2a (N2a) cells transfected with WT or KQ tau. n=3

independent experiments. (**i**) Additional characterization of the autophagosomes (APG) and autolysosomes (AUT) used in the study presented in main Figure 1h,i. A second antibody for LC3 that preferentially recognized LC3-II was used. n=3 mice. All values are mean+s.e.m. Differences were significant with untreated (- or None) for *p<0.05. For clarity purposes, only relevant statistical comparisons are presented. Uncropped blots are shown in Supplemental Figure 8. Source data are provided as a Source Data file.

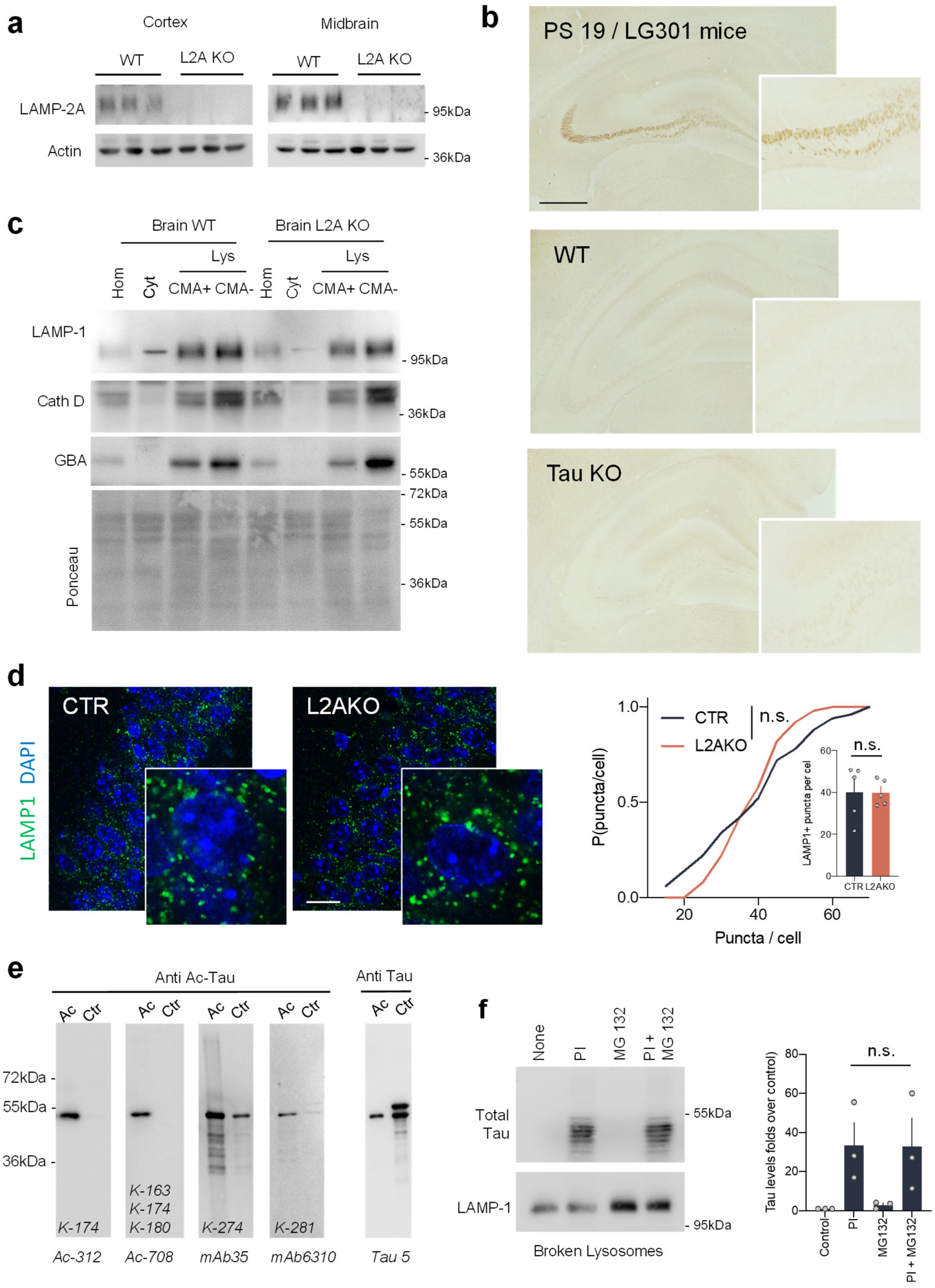

Supplemental Figure 2. Immunohistochemistry for acetylated tau in mouse brain sections. (**a**) Immunoblot for LAMP2A of the indicated brain regions from wild-type (WT) and L2A knock-out (L2AKO). n=3 mice per genotype. (**b**) Immunohistochemistry staining for acetylated tau of brain sections from PS19/LG301 (positive control), wild-type (WT) and tau knock out (negative control) mice. n=3 mice per genotype. Scale bar: 100µm. (**c**) Characterization of the CMA active (+) and inactive (-) lysosomes isolated from brains of WT and L2AKO mice and used for the analysis of associated tau proteins shown in Fig. 2b.

n=3 mice per genotype. (**d**) Immunofluorescence for LAMP1 in hippocampus of WT and L2AKO mice at 6months of age. Left: Representative images. Insets show higher magnification images. Nuclei are highlighted with DAPI. Scale bar: 20µm. Right: quantification of the number of puncta per cell shown as the cumulative distribution for individual cells (10 cells per animal per genotype) and mean per animal as inset. No significant (n.s.) statistical differences were detected between both genotypes using two-tailed t-tests. (**e**) Characterization of the lysins acetylated in the recombinant protein with antibodies specific for the residues. The lysine residues recognized for each antibody are annotated in the figure. The antibody against K-274 was not used in the in vitro assays with the recombinant protein as it presented some cross-reactivity with the control non-acetylated tau. n=3 independent experiments. (**f**) Incubation of disrupted lysosomes with recombinant tau in the presence of the indicated protease inhibitors (PI: cocktail of serine, cysteine and aspartic protease inhibitors; MG-132: proteasome inhibitor). Left: representative immunoblot. Right: densitometric quantification of 3 independent experiments. No significant (n.s.) statistical differences were detected between control and MG132 samples using two-tailed t-tests. Values are mean+s.e.m. Uncropped blots are shown in Supplemental Figure 8. Source data are provided as a Source Data file.

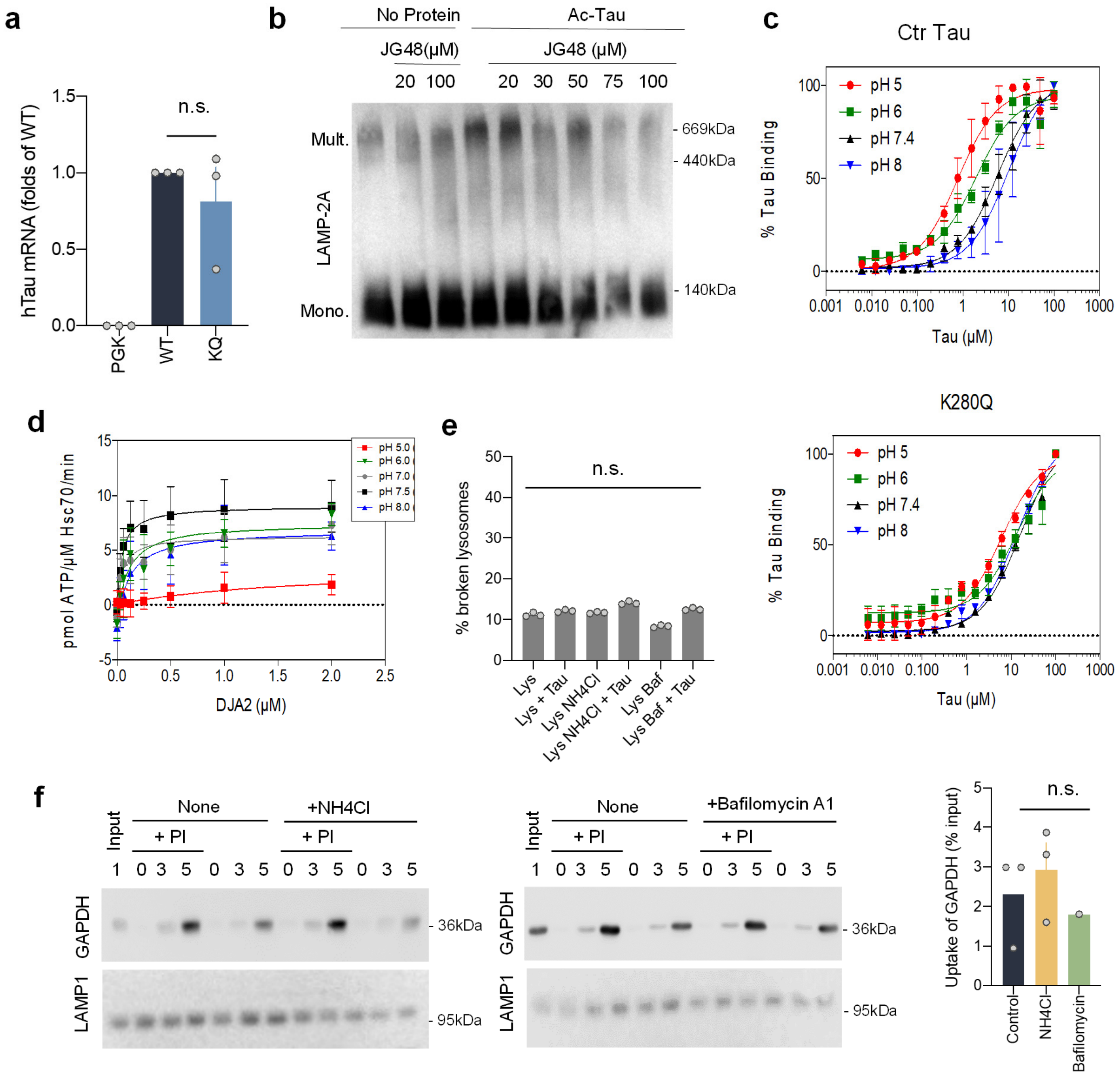

Supplemental Figure 3. Impact of acetylated tau and pH changes on CMA dynamics. (**a**) mRNA levels of human tau in N2a cells transfected with an empty vector (PGK) or plasmids coding for unmodified wild-type Tau (WT) or acetylation-mimetic K^{274,281}Q tau (KQ) tau. [One-way ANOVA $F_{(2,6)}$=16.87, p=0.0034] n=3 independent experiments. (**b**) Immunoblot for LAMP-2A of blue-native electrophoresis of lysosomes incubated alone or with in vitro acetylated tau (Ac-tau) and supplemented with the indicated concentrations of the hsc70 activator (JG48). n=3 independent experiments. (**c**) Full curves of tau or K^{280}Q tau binding assay to immobilized hsc70 determined by ELISA at different pH. n= 6 (in 2 independent experiments). (**d**) ATPase competency assay for hsc70 at different pH determined by absorbance to determine phosphate concentration; n= 6 (in 2 independent experiments). in triplicates. All values are mean+s.e.m. (**e**) Percentage of broken lysosomes after the indicated treatments calculated from the activity of hexosaminidase detected outside lysosomes relative to that in lysosomes. n= 3. No significant (n.s.) statistical differences were detected across conditions using One-way ANOVA. (**f**) Immunoblots of lysosomes from starved rat livers, pre-treated or not with protease inhibitors (PI) and/or NH_4Cl 20mM or Bafilomycin (20 μM) for 10 minutes at 4ºC and then incubated with GAPDH. Inpt: input 1 μg GAPDH. Right: Quantification of GAPDH uptake by the lysosomes shown on the right. n= 3. No significant (n.s.) statistical differences were detected across treatments using one-way ANOVA. Uncropped blots are shown in Supplemental Figure 8. Source data are provided as a Source Data file.

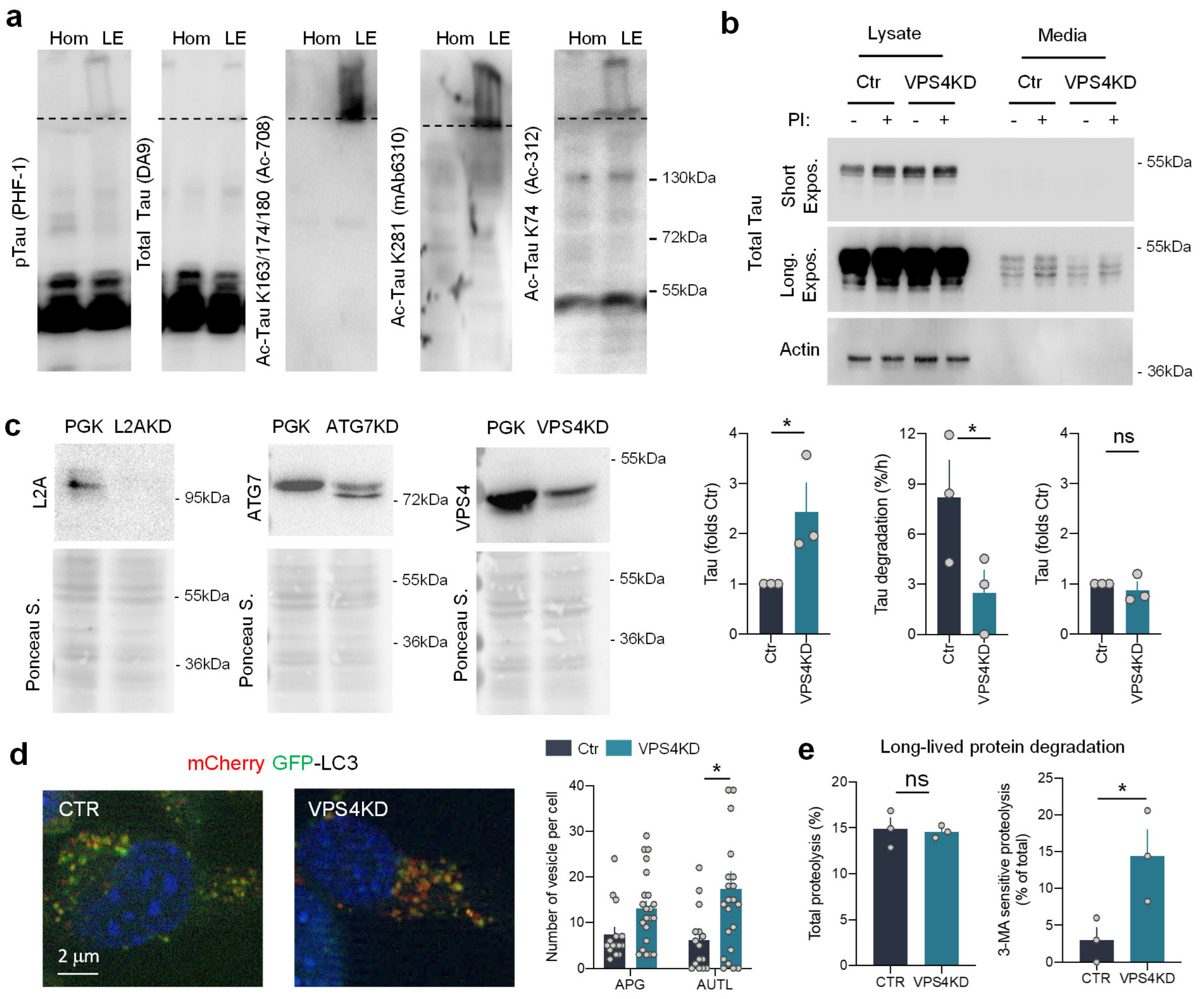

Supplemental Figure 4. siVps4-mediated knock down and Isolated lysosomes proteolytic activity. (**a**) Immunoblot with the indicated antibodies of homogenate (Hom) and late endosomes (LE) isolated from mice brain. Dashed line indicates separation between running and stacking gel. The same homogenate and LE fractions were loaded in triplicate in a gel and strips of the membrane were separately incubated with each of the antibodies to allow for comparison among antibodies. n=3 mice. (**b**) Immunoblots for the indicated proteins of lysate and media from control and siRNA-mediated Vps4 knock-down (VPS4KD) HEK cells transfected with FLAG-wild-type tau (WT tau). Top: representative immunoblot. Bottom: quantification of the amount of tau [Two tailed t-test t_4=6.708, p=0.0026] (left), percentage of tau degradation per hour [Two tailed t-test t_4=14.48, p=0.0001] (middle) and levels of extracellular tau [Two tailed t-test t_4=1.789, p=0.1481] (right) relative to those in control cells. n= 3 independent experiments. Values are mean+s.e.m. (**c**) Immunoblots for the indicated proteins to confirm the knock-down in the cells used for the experiments in main Figure 4g. n=3 independent experiments. (**d,e**) Analysis of macroautophagy activity in N2a cells control (CTR) or upon knock-down of Vps4 (VPS4KD). (**d**) Macroautophagy flux measured upon expression of the mCherry-GFP-LC3 tandem reporter. Left: Representative images of merged channels. Nuclei are highlighted with DAPI. Right: Quantification of number of autophagosomes [Two tailed t-test t_{35}=2.253, p=0.0307, n=15 (control) and 22 (VPS4KD) cells] (APG, yellow puncta) and autolysosomes (AUTL, red only puncta) [Two tailed t-test t_{35}=2.385, p=0.0226, n=15 (control) and 22 (VPS4KD) cells]. (**e**) Proteolysis of long-lived proteins in WT and VPS4KD cells radiolabelled with ^{3}H-leucine for 48h and maintained without additions or in the presence of 3-methyladenine (3MA) to inhibit macroautophagy. Protein degradation was measured as the amount of acid-precipitable radioactivity (amino acids and small peptides) released into

the media and values are expressed as percentage of total radiolabelled protein at the beginning of the chase. Left: Total protein degradation. Right: Percentage of total proteolysis sensitive to 3MA. n = 3 different experiments with triplicate wells. All values are mean+s.e.m. Differences with CTR were significant for *p<0.05, **p<0.01. For clarity purposes, only relevant statistical comparisons are presented. Uncropped blots are shown in Supplemental Figure 8. Source data are provided as a Source Data file.

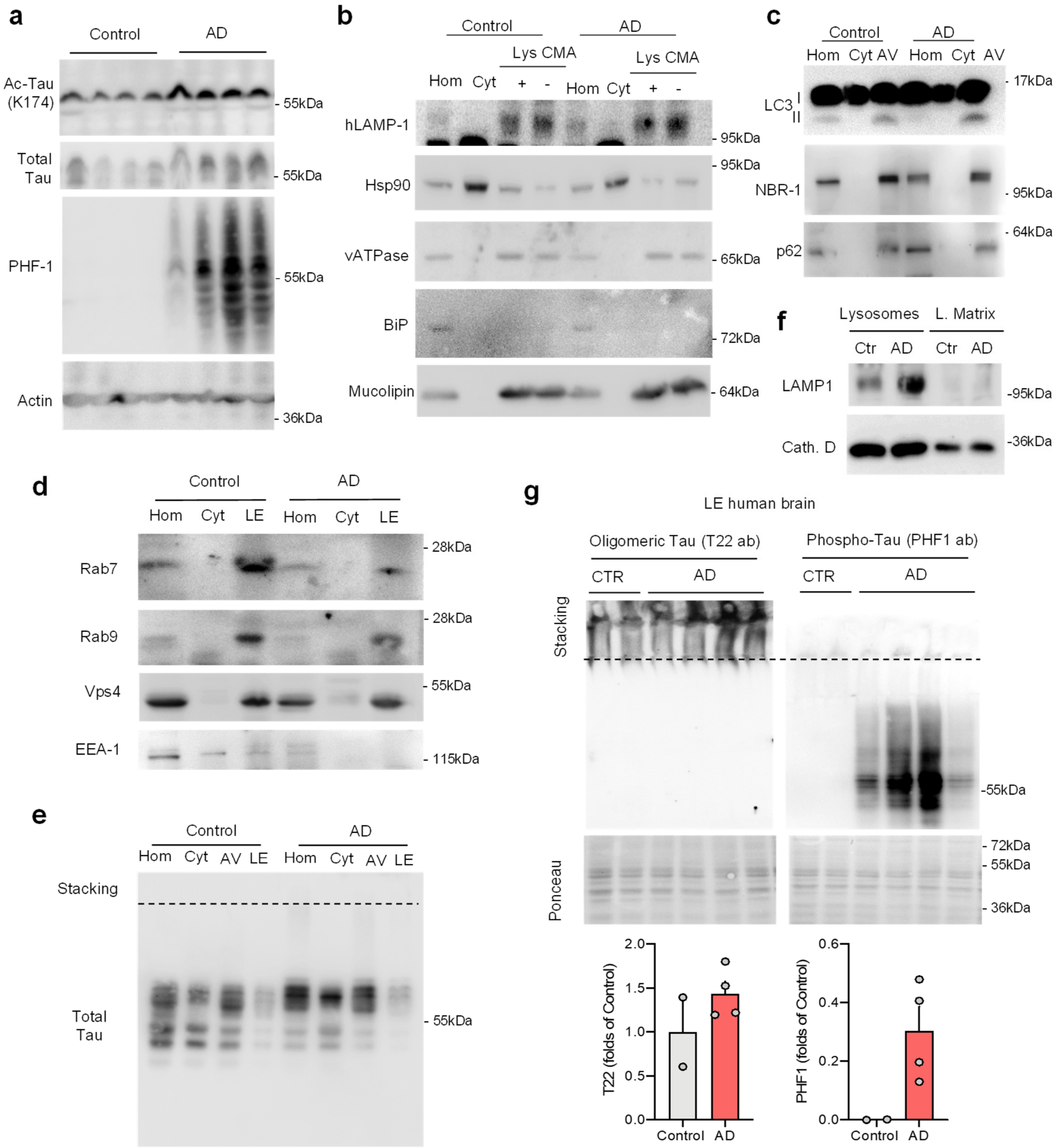

Supplemental Figure 5. Isolation of autophagic compartments from human brain. (**a**) Immunoblot for the indicated forms of tau in homogenates of brains from control and Alzheimer's disease (AD) patients. n=4 brains from each diagnosis are shown. (**b-e**) Immunoblot for the indicated proteins of homogenates (Hom), cytosol (Cyt) and two populations of lysosomes (Lys) with different CMA activity (CMA+ and CMA-) (**b**), or autophagic vacuoles (AV) (**c, e**) or late endosomes (LE) (**d, e**) isolated from the frontal area of brains from patients with Alzheimer's Disease (AD, n=6) and non-neurological patients of matching age (Control, n=7). (**f**) Immunoblot for the indicated proteins in total lysosomes and lysosomal matrix (L. Matrix) from AD patients and controls (Ctr). n=3 samples (1 per brain) per condition. (**g**) Immunoblot for oligomeric (T22 antibody) and phosphorylated (PHF1 antibody) human tau in LE isolated from the frontal area of brains from CTR or AD patients. Ponceau staining as control of loading of equal protein amounts per lane. Bottom: quantification of oligomeric (left) and phosphorylated (right) tau in CTR and AD patients. n= 2 non-neurological controls and 4 AD patients. Individual values and mean+s.e.m. are shown. No significant (n.s.) statistical differences were detected between both genotypes using two-tailed t-tests. Uncropped blots are shown in Supplemental Figure 8. Source data are provided as a Source Data file.

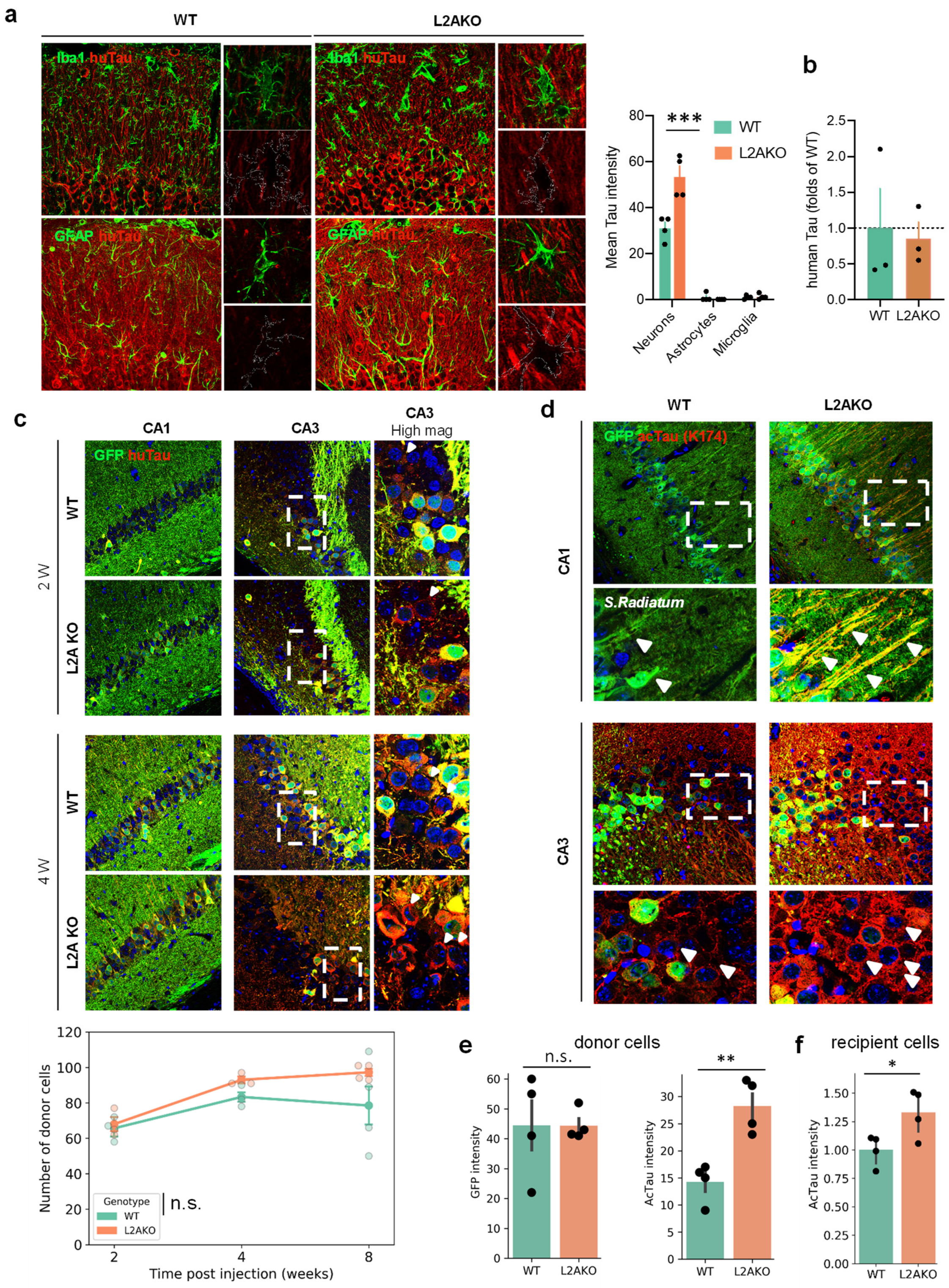

Supplemental Figure 6. CMA deficiency and Tau spreading. (**a**) Co-localization of human tau (huTau) with glia markers: microglia (Iba1) and astrocytes (GFAP) between WT and L2AKO mice at 8 weeks after injection. Right: quantification of mean huTau intensity in different brain cell populations. [Two way ANOVA Cell-type effect $F_{2,18}$=235.8, p<0.0001; Genotype effect $F_{1,18}$=16.26, p=0.0008], n= 4 per genotype. (**b**)

mRNA levels of human tau from brains of WT and L2AKO mice at 2 weeks after surgery [Two-tailed t-test t_4=0.2463, p=0.8176]. (**c**) Representative images of GFP and huTau immunofluorescence staining in CA1 and CA3 of the hippocampus between WT and L2AKO mice at 2 and 4 weeks after surgery. White arrows highlight recipient cells. Bottom: Number of donor cells overtime between WT and L2AKO mice; [Two-way ANOVA Cell-type effect $F_{2,14}$=5.510, p=0.0172] n= 3 mice per genotype for 2 and 4 weeks and 4 mice per genotype for 8 weeks). (**d**) Representative images of GFP and acetylated tau (K174) immunostainings in CA1 and CA3 of the hippocampus of WT and L2AKO mice at 8 weeks post-injection. (**e**) Quantification of GFP (left) and acetylated tau (Ac-Tau) (right) intensity in donor cells of WT and L2AKO mice at 8 weeks post-surgery [Two-tailed t-test t_6=4.5522, p=0.0039]; n=4 per genotype. (**f**) Quantification of acetylated tau (Ac-Tau) intensity in recipient cells of WT and L2AKO mice at 8 weeks post-surgery [Two-tailed t-test t_6=2.6239, p=0.0393]; n= 4 per genotype. Data are mean±s.e.m. Differences with WT mice were significant for **p<0.005, *** p<0.0005. Source data are provided as a Source Data file.

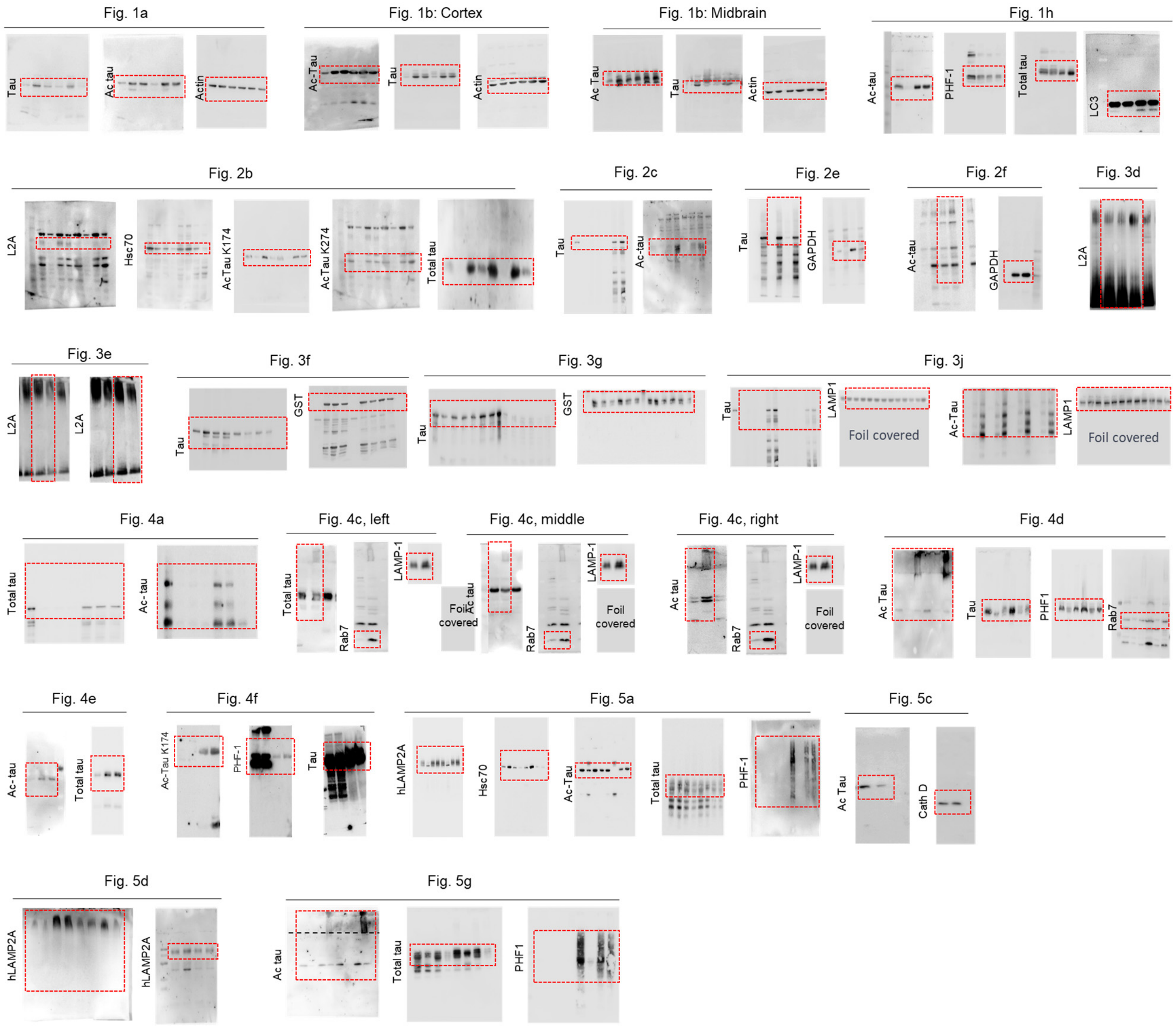

Supplementary Fig. 7. Uncropped gels from Main Figures 1-5. Dotted red boxes mark cropped areas shown in the main figure of the membrane blotted for the protein indicated on the left. Ponceau staining is shown in those instances in which total protein per line was used for normalization in the densitometric analysis.

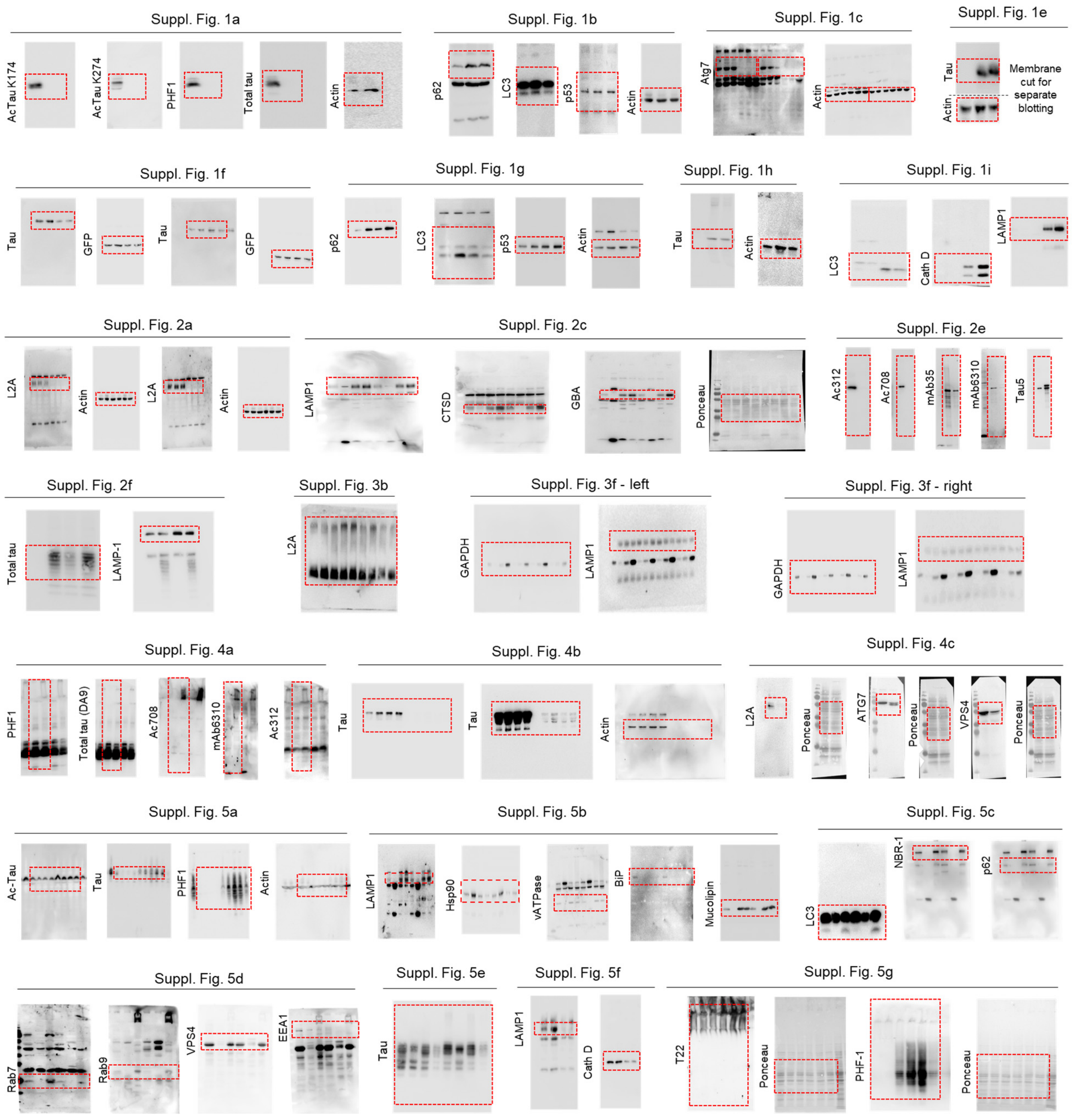

Supplementary Fig. 8. Uncropped gels from Supplemental Figures 1-6. Dotted red boxes mark cropped areas shown in the supplemental figure of the membrane blotted for the protein indicated on the left. Ponceau staining is shown in those instances in which total protein per line was used for normalization in the densitometric analysis.

Supplementary Table 1. qRT-PCR primers used in the study

Target	Forward primer	Reverse primer
human MAPT	5'-AGA-AGC-AGG-CAT-TGG-AGA-C-3'	5'-TCT-TCG-TTT-TAC-CAT-CAG-CC-3'
ACTB	5'AAG-GAC-TCC-TAT-AGT-GGG-TGA-CGA-3'	5'-ATC-TTC-TCC-ATG-TCG-TCC-CAG-TTG-3'
B2M	5'-CAC-TGA-ATT-CAC-CCC-CAC-TGA-3'	5'-TCA-CAT-GTC-TCG-ATC-CCA-GT-3'
