# Supplementary material for: Acetylated tau inhibits chaperone-mediated autophagy and promotes tau pathology propagation in mice
Source: Nat Commun. 2021 Apr 14;12:2238. doi: 10.1038/s41467-021-22501-9 (PMC8047017; doi:10.1038/s41467-021-22501-9)
Supplement: Supplementary file 2 — Reporting Summary [file 41467_2021_22501_MOESM2_ESM.pdf]

nature research

Corresponding author(s): Ana Maria Cuervo

Last updated by author(s): 01/25/2021

Reporting Summary

Nature Research wishes to improve the reproducibility of the work that we publish. This form provides structure for consistency and transparency in reporting. For further information on Nature Research policies, see our Editorial Policies and the Editorial Policy Checklist.

Statistics

For all statistical analyses, confirm that the following items are present in the figure legend, table legend, main text, or Methods section.

n/a	Confirmed	
☐	☒	The exact sample size (*n*) for each experimental group/condition, given as a discrete number and unit of measurement
☐	☒	A statement on whether measurements were taken from distinct samples or whether the same sample was measured repeatedly
☐	☒	The statistical test(s) used AND whether they are one- or two-sided<br>*Only common tests should be described solely by name; describe more complex techniques in the Methods section.*
☒	☐	A description of all covariates tested
☐	☒	A description of any assumptions or corrections, such as tests of normality and adjustment for multiple comparisons
☐	☒	A full description of the statistical parameters including central tendency (e.g. means) or other basic estimates (e.g. regression coefficient) AND variation (e.g. standard deviation) or associated estimates of uncertainty (e.g. confidence intervals)
☐	☒	For null hypothesis testing, the test statistic (e.g. F, t, r) with confidence intervals, effect sizes, degrees of freedom and P value noted<br>*Give P values as exact values whenever suitable.*
☒	☐	For Bayesian analysis, information on the choice of priors and Markov chain Monte Carlo settings
☒	☐	For hierarchical and complex designs, identification of the appropriate level for tests and full reporting of outcomes
☒	☐	Estimates of effect sizes (e.g. Cohen's *d*, Pearson's *r*), indicating how they were calculated

Our web collection on statistics for biologists contains articles on many of the points above.

Software and code

Policy information about availability of computer code

Data collection	Images were acquired with a Leica confocal TCS-SP8 (Leica Microsystem) using the Leica LAS-X software for image adquisition and ImageJ Software (v.2.1.0) (NIH) was used for image processing and analysis.
Data analysis	All software packages used in this study are listed in the "Statistical Analysis" section of the Online Methods . Basic data handling was done in Microsolft Excel 365 (v.2101). Data analysis was performed with Prism software (v9 - Graph Pad Software Inc) or using Python (Python software foundation v.3.7.4 available at https://www.python.org/) and the scientific python stack: scipy (v.1.3.1), numpy (v.1.17.2), and matplotlib (v.3.1.1). Image analysis and quantification was performed using ImageJ (v.2.1.0).

For manuscripts utilizing custom algorithms or software that are central to the research but not yet described in published literature, software must be made available to editors and reviewers. We strongly encourage code deposition in a community repository (e.g. GitHub). See the Nature Research guidelines for submitting code & software for further information.

Data

Policy information about availability of data

All manuscripts must include a data availability statement. This statement should provide the following information, where applicable:

- Accession codes, unique identifiers, or web links for publicly available datasets
- A list of figures that have associated raw data
- A description of any restrictions on data availability

There are not restrictions on data availability in this manuscript. Source data are provided with this paper. All the information is included in the manuscript has been included in the Source data table. This table is organized by Figure (main and supplemental) and inside each figure sheet by panels and it includes raw data and full statistic report.

Field-specific reporting

Please select the one below that is the best fit for your research. If you are not sure, read the appropriate sections before making your selection.

- [x] Life sciences
- [ ] Behavioural & social sciences
- [ ] Ecological, evolutionary & environmental sciences

For a reference copy of the document with all sections, see nature.com/documents/nr-reporting-summary-flat.pdf

Life sciences study design

All studies must disclose on these points even when the disclosure is negative.

Sample size	In all instances "n" refers to individual experiments. All the experiments were performed at least 3 times, unless otherwise stated.<br>For the studies in life animals the number of animals per group was determined through power analysis using the information from similar studies from our group or collaborators with similar methods (PMID: 31249873, PMID: 29024336; PMID: 23455607; PMID: 15333840)<br>For the studies performed in brain slices, cells lines in culture and in vitro assays we determine number of experimental repetitions to account for technical variability and changes in culture conditions based on our previous studies using those systems (PMID: 31249873, PMID: 29024336; PMID: 23455607; PMID: 15333840)<br>For the studies using isolated organelles from animals, the number of specimen used was determined based on the average values of enrichment and recovery for the specific fraction using endogenous markers for each compartment from our previous studies. (PMID: 16917501; PMID: 9038169; PMID: 15333840)
Data exclusions	No data exclusion was done.
Replication	Every experiment was performed in at least 3 independent replicates. Experiments in cells in culture or those involving isolation of intracellular organelles, were performed in different days to confirm reproducibility of the procedures. In addition recovery and enrichment for each subcellular fractionation experiment was calculated to compare purity and efficiency of isolations done in different days. All independent replications were successful.
Randomization	For the studies involving live animals, animals were randomly attributed to each surgery group according to their genotype using the "SELECT BETWEEN RANGE" function in Microsoft Excel.<br>For the studies involving cells in culture or in vitro assays with purified orgenelles, treatment groups were attributed randomly between wells and plates to account for well or tube positioning effects.
Blinding	Immunofluorescence/immunohistochemistry of protein levels were performed blinded to their genotype. Immunofluorescence and the quantification of number of puncta was performed blinded to the experimental group. For the morphometric analyses of the electron microscopy samples, annotated micrographs were performed blinded. For in vivo experiments, samples were blinded to the experimenter for the whole experiment, image acquisition and data analysis. Blinding was lifted only for the final statistical analysis.

Reporting for specific materials, systems and methods

We require information from authors about some types of materials, experimental systems and methods used in many studies. Here, indicate whether each material, system or method listed is relevant to your study. If you are not sure if a list item applies to your research, read the appropriate section before selecting a response.

Materials & experimental systems

n/a	Involved in the study	
[ ]	[x]	Antibodies
[ ]	[x]	Eukaryotic cell lines
[x]	[ ]	Palaeontology and archaeology
[ ]	[x]	Animals and other organisms
[ ]	[x]	Human research participants
[x]	[ ]	Clinical data
[x]	[ ]	Dual use research of concern

Methods

n/a	Involved in the study	
[x]	[ ]	ChIP-seq
[x]	[ ]	Flow cytometry
[x]	[ ]	MRI-based neuroimaging

Antibodies

Antibodies used	rat anti-mouseLAMP1, Hybridoma bank #1D4B, 1:5000<br>mouse anti-humanLAMP1, Hybridoma bank #H4A3, 1:5000<br>mouse anti-LAMP2, Hybridoma bank #H4B4, 1:5000<br>rabbit anti-humanLAMP2A, abcam #ab18528, 1:1000<br>mouse anti-GAPDH, abcam #ab8245, 1:1000<br>mouse anti-tau [human - Tau13], abcam #ab19030, 1:1000

goat anti-Iba1, abcam #ab5076, 1:1000
rabbit anti-GFAP, abcam #ab5804, 1:1000
mouse anti-GST, Invitrogen #136700, 1:1000
rabbit anti-actin, Sigma-Aldrich #A2066, 1:5000
mouse anti-FLAG, Sigma-Aldrich #F4042, 1:1000
rabbit anti-VPS4, Sigma-Aldrich #sab4200025
rabbit anti-tau [Clone T22], Sigma-Aldrich #ABN454, 1:1000
rat anti-Hsp90, Stressgen #adi-spa-835-f, 1:1000
rabbit anti-LC3B, Cell Signaling #2775, 1:1000
mouse anti-p53, Cell Signaling #2524s, 1:1000
rabbit anti-Rab9, Cell Signaling #5133, 1:1000
rabbit anti-Atg7, Cell Signaling #2631, 1:1000
mouse anti-eGFP, Origene #ta150052, 1:1000
mouse anti-Hsc70 [Clone 13D3], Novus Biologicals #NB120-2788, 1:1000
rabbit anti-Atg5, Novus Biologicals, #NB110-53818, 1:1000
mouse anti-NBR1, Abnova #H00004077-B01P, 1:1000
rabbit anti-p62, Enzo Life Science #BML-PW9860-0100, 1:2000
rabbit anti-tau [human - TAUY9], Enzo Life Science #BML-TA3119-0100, 1:1000
mouse anti-BiP, BD Transduction #610978, 1:1000
mouse anti-EEA1, BD Biosciences #610456, 1:1000
rabbit anti-tau, Santa Cruz, #sc-5587, 1:2000
rabbit anti-LAMP2A, custom antibody (A.M. Cuervo)
mouse anti-tau [PHF-1], custom antibody (P. Davies)
mouse anti-tau [DA9], custom antibody (P. Davies)
mouse anti-AcetylatedTau-K174 [AC312], custom antibody (L. Gan)
mouse anti-AcetylatedTau-K274, custom antibody (L. Gan)

Validation

We have provided catalog numbers or clone numbers for all the commercial antibodies in the Method section . Most antibodies used are commercial and have been chosen based on extensive use in the literature. When available antibodies where validated using cell lines knock-down for the antibody target. For non commercial antibodies generated in our laboratories, they have all been extensively validated with positive controls (i.e in the case of antibodies against tau and different acetylated tau variants mouse models or cell lines expressing human tau were used; in the case of antibodies against LAMP-2A using cell lines expressing recombinant LAMP2A) and negative controls (tissues from tau knock out mice or from LAMP-2A knock out mice). In addition, we have openly shared these antibodies with the scientific community who has validated them in multiple publications. See examples in PMID: 31906970; PMID: 27041503; PMID: 26390242; PMID: 20869593.

Eukaryotic cell lines

Policy information about cell lines

Cell line source(s)

The mouse neuroblastoma cell line Neuro-2a (N2a) was a gift from Drs. Mandelkow (DZNE, Germany), mouse embryonic fibroblasts (MEFs) from wild-type (WT) or Atg5-/- (KO) mice were a gift from Dr. N. Mizushima (The University of Tokyo). MEFs from wild-type (WT) mice were generated in our laboratory PMID: 25620427.

Authentication

All the members of our laboratories are trained on the Best Laboratory Practices and safe tissue culturing techniques to prevent contaminating the established cell lines with cells from foreign cell lines as describe on the ICLAC website. The Neuro-2a cell lines used in this project were authenticated by karyotyping at regular intervals to confirm their identity. For MEFs cells, we also validated the specific knock-out through qPCR and immunoblot.

Mycoplasma contamination

All the cells lines were tested for mycoplasma contamination using DNA staining protocol with Hoechst 33258 dye (weekly) or MycoSenser PCR Assay Kit (monthy). All cell lines used in the present study were free of such contamination.

Commonly misidentified lines
(See ICLAC register)

No commonly misidentified cell lines were used in the present study.

Animals and other organisms

Policy information about studies involving animals; ARRIVE guidelines recommended for reporting animal research

Laboratory animals

We listed the detailed information for the animals in the Method section "Animals and cells". Mice used for this study were 12 weeks old male C57BL/6 wild Type and LAMP-2A knock-out (L2AKO), ATG7 knockout (Atg7KO). Animals were maintained at 19-23°C 30-60% relative humidity and timer-controlled 12h light/dark cycle.

Wild animals

The study did not involve wild animals

Field-collected samples

The study did not involve samples collected from the field

Ethics oversight

All animal procedures were conducted under an animal study protocol approved by the Institutional Animal Care and Use Committee (IACUC) of the Albert Einstein College of Medicine as stated in Online Methods.

Note that full information on the approval of the study protocol must also be provided in the manuscript.

Human research participants

Policy information about studies involving human research participants

Population characteristics	All population demographics are detailed in Supplementary Table 1
Recruitment	Recruitment was not done as part of this study. Samples utilized were part of the human brain bank at the ADRC Neuropathology Core. Selection for the lysosomal isolation was done on the basis of the histopathology report that assigned brain tissue as "healthy control" or "AD pathology" and the postmortem time (within the reported range) in order to keep samples comparable. We are not aware of any bias on the selection of tissues that could impact the results presented.
Ethics oversight	The Washington University IRB reviewed the Knight ADRC Neuropathology Core (from where the brains were obtained) operating protocol as well as this specific study and determined it was exempt from approval. In the state of Missouri, individuals can give prospective consent for autopsy. Our participants provide this consent by signing the hospital's autopsy form. If the participant does not provide future consent before death the DPOA or next of kin provide it after death.

Note that full information on the approval of the study protocol must also be provided in the manuscript.
